# Telomere alterations in neurofibromatosis type 1-associated solid tumors

**DOI:** 10.1186/s40478-019-0792-5

**Published:** 2019-08-28

**Authors:** Fausto J. Rodriguez, Mindy K. Graham, Jacqueline A. Brosnan-Cashman, John R. Barber, Christine Davis, M. Adelita Vizcaino, Doreen N. Palsgrove, Caterina Giannini, Melike Pekmezci, Sonika Dahiya, Murat Gokden, Michael Noë, Laura D. Wood, Christine A. Pratilas, Carol D. Morris, Allan Belzberg, Jaishri Blakeley, Christopher M. Heaphy

**Affiliations:** 1 Departments of Pathology, Johns Hopkins University School of Medicine, Sheikh Zayed Tower, Room M2101, 1800 Orleans Street, Baltimore, MD 21231 USA; 2 Departments of Ophthalmology, Johns Hopkins University School of Medicine, Sheikh Zayed Tower, Room M2101, 1800 Orleans Street, Baltimore, MD 21231 USA; 3 Sidney Kimmel Comprehensive Cancer Center, Johns Hopkins University School of Medicine, Sheikh Zayed Tower, Room M2101, 1800 Orleans Street, Baltimore, MD 21231 USA; 4 Departments of Neurology, Johns Hopkins University School of Medicine, Sheikh Zayed Tower, Room M2101, 1800 Orleans Street, Baltimore, MD 21231 USA; 5 Department of Epidemiology, Johns Hopkins Bloomberg School of Public Health, Baltimore, MD USA; 6 Department of Laboratory Medicine and Pathology, Mayo Clinic, Rochester, MN USA; 7 Department of Pathology, University of California San Francisco, San Francisco, CA USA; 8 Department of Pathology and Immunology, Washington University, St. Louis, MO USA; 9 University of Arkansas, Little Rock, AR USA; 10 Department of Orthopedics, Johns Hopkins University School of Medicine, Baltimore, MD USA; 11 Department of Neurosurgery, Johns Hopkins University School of Medicine, Baltimore, MD USA

**Keywords:** NF1, ATRX, Alternative lengthening of telomeres, Glioma, MPNST

## Abstract

The presence of Alternative lengthening of telomeres (ALT) and/or ATRX loss, as well as the role of other telomere abnormalities, have not been formally studied across the spectrum of NF1-associated solid tumors. Utilizing a telomere-specific FISH assay, we classified tumors as either ALT-positive or having long (without ALT), short, or normal telomere lengths. A total of 426 tumors from 256 NF1 patients were evaluated, as well as 99 MPNST tumor samples that were sporadic or of unknown NF1 status. In the NF1-glioma dataset, ALT was present in the majority of high-grade gliomas: 14 (of 23; 60%) in contrast to only 9 (of 47; 19%) low-grade gliomas (*p* = 0.0009). In the subset of ALT-negative glioma cases, telomere lengths were estimated and we observed 17 (57%) cases with normal, 12 (40%) cases with abnormally long, and only 1 (3%) case with short telomeres. In the NF1-associated malignant nerve sheath tumor (NF1-MPNST) set (*n* = 75), ALT was present in 9 (12%). In the subset of ALT-negative NF1-MPNST cases, telomeres were short in 9 (38%), normal in 14 (58%) and long in 1 (3%). In the glioma set, overall survival was significantly decreased for patients with ALT-positive tumors (*p* < 0.0001). In the NF1-MPNST group, overall survival was superior for patients with tumors with short telomeres (*p* = 0.003). ALT occurs in a subset of NF1-associated solid tumors and is usually restricted to malignant subsets. In contrast, alterations in telomere lengths are more prevalent than ALT.

**Electronic supplementary material:**

The online version of this article (10.1186/s40478-019-0792-5) contains supplementary material, which is available to authorized users.

## Introduction

Patients with neurofibromatosis type 1 (NF1) are prone to develop a variety of neoplasms [4]. The most common central nervous system (CNS) tumors in these patients are pilocytic astrocytomas (PA) which frequently involve the optic pathways. However, other CNS tumor types may also develop [38] (reviewed in Nix et al. [29]). The specific genetic drivers of these tumors are just beginning to be characterized, in contrast to gliomas developing in a sporadic setting, for which more information is known [3].

Telomeres consist of a repetitive hexameric DNA sequence (TTAGGG_n_) bound by the shelterin protein complex. Since telomeres progressively shorten with each division cycle, cancer cells require activation of a telomere maintenance mechanism. The predominant mechanism is the upregulation of telomerase, which may be accomplished by numerous genetic means [2, 19]. In contrast, a subset of cancers use the telomerase-independent *Alternative* *Lengthening of* *Telomeres* (ALT) [13] mechanism, which is mediated by homology directed repair. These unique ALT-associated features include the presence of ultra-bright telomeric foci, dramatic cell-to-cell and intracellular telomere heterogeneity, and the presence of single stranded extrachromosomal circles containing the C-rich telomere repeat sequence (C-circles). Even in the absence of ALT, variations in telomere length are being increasingly recognized as a prognostic factor in cancer [14, 41].

Prior studies have related alterations in the *alpha thalassemia/mental retardation syndrome X-linked (ATRX), death domain-associated protein (DAXX), or SWI/SNF related, matrix associated, actin dependent regulator of chromatin (SMARCAL1)* genes with ALT in some cancers [9, 12]. *ATRX* mutations and ALT are associated with specific molecular subgroups of brain tumors [28]. We reported for the first time a high frequency of ATRX loss and ALT in high-grade and diffuse gliomas developing in NF1 patients [39], and more recent studies have also documented *ATRX* mutations in aggressive NF1-associated gliomas [8, 37]. Genomic studies have identified alterations in *SUZ12* and *EED* in the majority of malignant nerve sheath tumors (MPNST) [21, 43]. These mutations result in profound epigenetic alterations, including loss of H3K27 trimethylation [33].

While most MPNSTs maintain their telomeres presumably through increased telomerase activity [25], recent studies have demonstrated abnormally short telomeres in MPNSTs, in contrast to neurofibromas [18]. Conversely, a global study of telomere lengths across 6835 tumors (31 tumor types) documented that two tumor types in particular, glioma and sarcomas, are characterized by longer telomeres [34]. In the current study, we interrogate the full spectrum of NF1-associated solid tumors for telomere alterations and possible associations with clinicopathologic characteristics and outcomes.

## Materials and methods

### Patients and tumor samples

A total of 426 tumor samples from 256 NF1 patients were studied. NF1 status was based on established clinical consensus criteria [27], with various features abstracted from the clinical records. In addition, a set of 99 MPNST samples (18 non-syndrome associated, 2 schwannomatosis-associated, and 79 of unknown NF1 status) from 81 patients were also studied. All histologic sections were reviewed by a board certified neuropathologist (FJR) and classified to the extent feasible using criteria embodied in the 2016 WHO Classification of Tumors of the Central Nervous System [23] and recently proposed criteria for nerve sheath tumors [26, 30]. Tumor samples included whole sections (*N* = 364) or sections obtained from 3 different TMAs (*N* = 161) consisting of NF1-associated gliomas and MPNSTs that have been previously characterized [24, 32, 36]. All ethical standards were followed and the study was performed under IRB approval, with consent or waiver of consent approval.

### Telomere-specific FISH and CISH

ALT status was performed and interpreted using previously published criteria and was characterized by the presence of distinct large telomeric DNA signals using fluorescence in situ hybridization (FISH) [12, 13, 39] or chromogenic in situ hybridization (CISH) [39] assays. In brief, tissue slides were deparaffinized, hydrated, and steamed for 25 min in citrate buffer (Vector Laboratories). This was followed by dehydration and hybridization with a Cy3-labeled peptide nucleic acid (PNA) probe complementary to the mammalian telomere repeat sequence [(N-terminus to C-terminus) CCCTAACCCTAACCCTAA]. In the FISH approach, an Alexa Fluor-488–labeled PNA probe specific to human centromeric DNA repeats was also included as a control to assess the validity of the hybridization. Following post-hybridization washes, the slides were counterstained with DAPI following post-hybridization washes. The telomere-specific CISH was previously outlined. Briefly, deparaffinized slides were hydrated, steamed for 25 min in citrate buffer, dehydrated, and hybridized with a Cy3-labeled PNA probe (see above). Sections were blocked against endogenous peroxidase activity with Dual Endogenous Enzyme-Blocking Agent (Dako) for 10 min, incubated with a monoclonal anti-Cy3/Cy5 antibody (ab52060, Abcam, 1:2500) for 1 h at room temperature, then incubated with an anti-mouse secondary antibody (Leica Microsystems) for 30 min, and detected with 3,30-diaminobenzidine (Sigma-Aldrich) after 10 min. Finally, sections were counterstained with hematoxylin, rehydrated, and mounted for visual inspection. In the subset of ALT-negative cases with appropriate hybridization signals in the internal non-neoplastic components, telomere lengths were qualitatively scored by direct visual assessment of stained slides comparing telomere signals from tumor cells with telomere signals from benign cells (entrapped non-neoplastic cells) from the same case. In all cases, signals from benign cells were considered 2+ (normal). Telomere signals in tumor cells of different cases ranged from short (1+) to normal (2+) to long (3+).

### Immunohistochemistry

Immunohistochemical studies that were previously performed as part of the clinical diagnostic workup were reviewed. However, in most cases immunohistochemical studies were systematically performed using antibodies recognizing either ATRX, DAXX, or H3K27me3.

Immunohistochemical studies were systematically performed in most cases using an ATRX antibody (Rabbit polyclonal, 1:200 dilution, catalog# HPA001906 Sigma-Aldrich). Immunostaining was performed on automated instruments (BenchMark, Ventana Medical Systems, Tucson, AZ, USA). The immunohistochemical protocol included deparaffinization, hydration, antigen retrieval, primary antibody incubation, and detection and visualization as per manufacturer’s instructions. Immunohistochemistry for DAXX (Rabbit polyclonal, 1:100 dilution, catalog# HPA008736, Atlas Antibodies) was performed manually. Sections were incubated with primary antibody for 2 h at room temperature followed by secondary antibody (Leica Microsystems) for 30 min and detected with 3,30-diaminobenzidine (Sigma-Aldrich) after 10 min. Immunohistochemistry for H3K27me3 was performed manually in whole tissue sections using a mouse monoclonal antibody (Abcam, Cat# ab6002, Cambridge, MA) at 1:1600 dilution an incubated overnight at 4 °C. The subset of MPNST cases studied in a TMA were tested for H3K27me3 status as part of a previous study [32]. Immunoreactivity preservation in internal non-neoplastic cell components was required for valid interpretation in all cases to evaluate ATRX, DAXX, or H3K27me3 loss. In cases where the loss was clearly limited to a subset of neoplastic cells, with preserved reactivity in non-neoplastic components, the loss was interpreted as “partial”.

### Next generation sequencing

To identify possible somatic genetic alterations associated with ALT, we performed next generation sequencing on the 9 ALT-positive tumors with available tissue (6 gliomas and 3 MPNST). Adjacent formalin-fixed, paraffin-embedded sections cut at 5-μm thick were scrapped into 2 ml tubes for DNA extraction, focusing on areas enriched for neoplastic cells. An automated Siemens Tissue Preparation System (Siemens Healthcare Diagnostics, Inc., Tarrytown, NY) was used for DNA extraction and purification. Quantification of Genomic DNA was performed using the Qubit 2.0 Fluorometer (Life Technologies, Carlsbad, CA). DNA libraries were prepared using Agilent SureSelect-XT reagents (Agilent Technologies, Inc., Santa Clara, CA). Genomic regions of interest were captured using an Agilent custom-designed bait set covering the full coding regions of 644 cancer associated genes, although exon 1 is poorly covered for some genes. Genes of interest included *NF1, SUZ12, EED, ATRX, DAXX, and BRAF*. Sequencing of libraries was achieved to an average unique read depth of greater than 500X using Sequencing by Synthesis (SBS) 2 × 100 base pairs (bp) paired-end cluster generation on the Illumina HiSeq 2500 plataform (Illumina, Inc., San Diego, CA). FASTQ files were generated from Binary Cluster Files (.bcl) using the Illumina bcl2fastq v1.8.4 software with parameters set as per vendor’s specifications. FASTQ files were aligned to the human genome reference hg19 (GRCh37) using the Burrows-Wheeler Aligner v0.7.10 algorithm with default settings. BAM files (.bam) were generated using Picard Tools v1.119 and variant calling was performed using in-house variant caller algorithm (MDLVC v5.0) cross referenced with HaplotypeCaller (Genome Analysis Tool Kit 3.3) under discovery mode in the coding regions of target genes. All variant calls were inspected using Integrated Genomics Viewer v2.3.4 (IGV; Broad Institute, MIT Harvard, Cambridge, MA) and annotated with dbSNP v150 and COSMIC v82 databases. Common single nucleotide polymorphisms (SNPs; population allele frequency > 50%) were excluded from analysis. Matched normal (tissue or peripheral blood) was not available for comparison. Mean target coverage of the full coding regions (including exon 1) of *ATRX, DAXX*, and *NF1* was 688 reads (330 to 930 average base read depth), 811 reads (406 to 1129 average base read depth), and 823 reads (423 to 1089 average base read depth), respectively, across all cases.

### Statistical analysis

All characteristics were described using proportions, ranges, means, medians, and standard deviations as appropriate. Proportions were compared using Chi-Square or Fisher’s exact tests as appropriate. Survival rates were visualized using Kaplan–Meier curves and separations of curves were assessed using the log-rank test. Overall survival was calculated from the time of first pathologic diagnosis to death. Recurrence-free survival was calculated from the time of first pathologic diagnosis to the first evidence of tumor growth per clinical record, or time of death. In further analyses in the glioma and MPNST cohorts, Cox-proportional hazard models were used to assess the independent effects of either telomere length or ALT on overall survival while adjusting for age and grade. Statistical analyses were performed using GraphPad Prism version 8.0 (San Diego, CA), SAS version 9.4 (Cary, NC), and R version 3.3.2.

## Results

### ALT and long telomeres are frequent in NF1-associated CNS tumors

A total of 78 CNS tumors from 70 patients with NF1 were analyzed, including 70 primary biopsies or resections and 8 surgeries/recurrences. Clinicopathologic and molecular features are outlined for all CNS tumors (Additional file 1: Table S1) and summarized for the gliomas in Table 1. In the glioma cohort, ALT was present in 23 (of 70; 32.9%) tumors. When comparing groups, ALT was present in the majority of high-grade gliomas 14 (of 23; 60%) compared to 9 (of 47; 19%) low-grade gliomas, a difference that was statistically significant (*p* = 0.0009, χ^2^ test). When focusing on different pathologic subtypes, ALT was present in only 2 (of 26, 8%) NF1-associated PA compared to 21 (of 44, 47%) other NF1-CNS (non-PA) tumors (*p* = 0.001, Fisher Exact Test). When assessing additional tumor samples/recurrences, there was a perfect concordance among multiple resections from the same patient, with ALT present in 3 and absent in 5 pathology samples from the same patient. Among 23 cases with ALT, ATRX loss was present in 17 (74%) (14 complete, 3 partial) and preserved in 6 (26%). In contrast, DAXX was preserved in 11 (of 11) cases. Finally, among the ALT-negative cases, 3 (of 42; 7%) cases tested demonstrated partial ATRX loss, while DAXX was preserved in 14 (of 14) cases.

**Table 1:** Clinico-pathologic and molecular features of gliomas, MPNST, and neurofibromas

	Gliomas	MPNST	Neurofibromas
	(*N* = 70)	(*N* = 152)	(*N* = 77)^a^
Mean age (SD)	20.6 (13.8)	38.9 (17.3)	30.6 (17.7)
Gender (%)			
Male	52.9	49.0	48.1
Female	47.1	51.0	52.0
Grade (%)			
High	32.9	88.7	–
Low	67.1	11.3	–
ALT status (%)			
Positive	32.9	16.6	–
Negative	67.1	81.5	–
Missing	–	2.0	–
Telomeres (%)			
Normal	24.3	9.9	–
Short	1.4	9.3	–
Long	17.1	0.7	–
ALT	32.9	16.6	–
Missing	24.3	63.6	–
NF status (%)			
NF1	–	61.6	–
Unknown	–	38.4	–

^a^Multiple samples tested per patient (77 patients with a mean of 2.4 samples per individual)

**Fig. 1:**
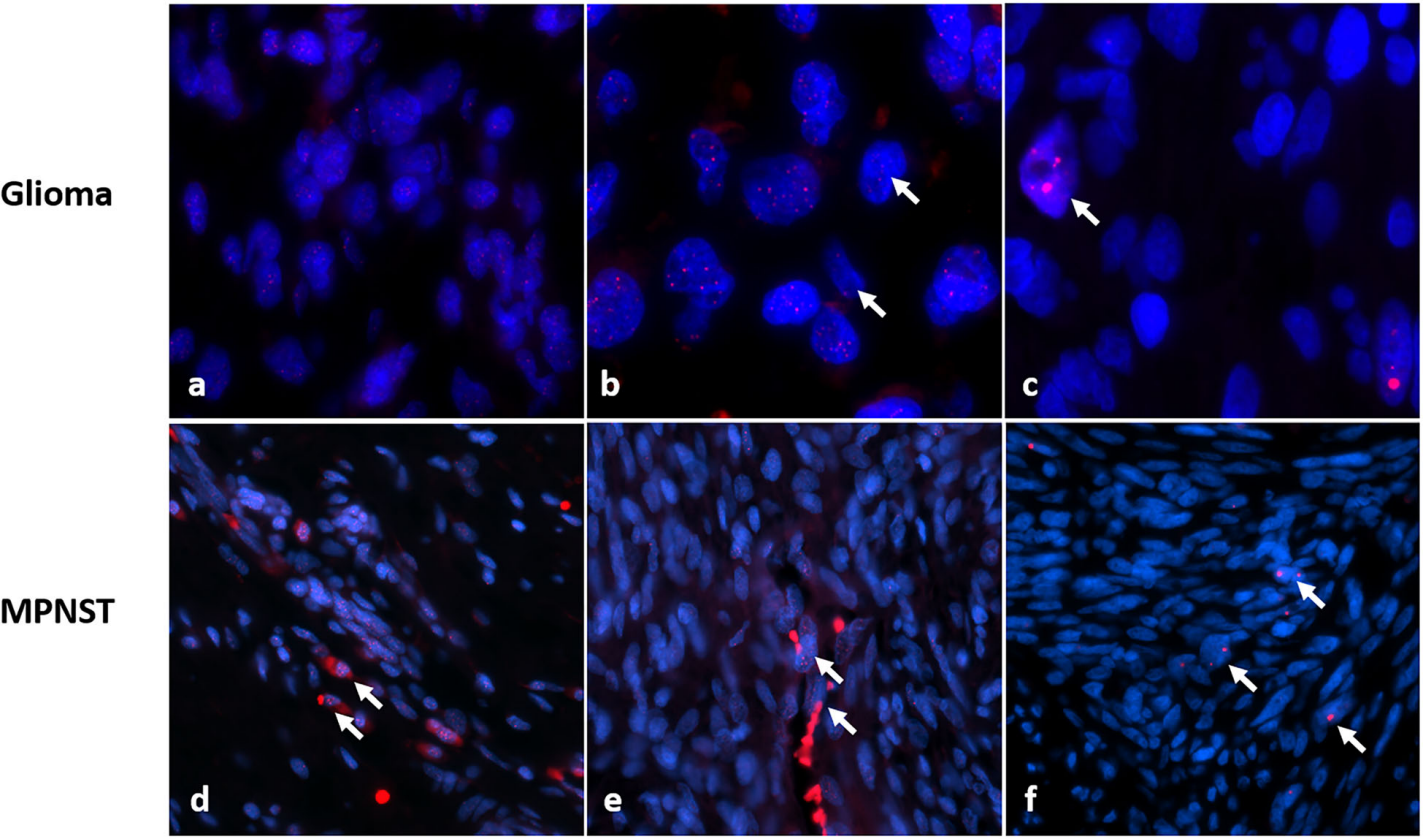
Telomere alterations in NF1-solid tumors. NF1-associated gliomas demonstrated telomeres of normal length (**a**), long (comparted to internal non-neoplastic cells, arrows) (**b**) or ALT (arrows) (**c**). ALT was relatively frequent in NF1-associated gliomas, while the majority of evaluable ALT negative gliomas had normal or abnormally long telomeres. In contrast, MPNST demonstrated telomeres of normal length (comparted to internal non-neoplastic cells, arrows) (**d**), short (comparted to internal non-neoplastic cells, arrows) (**e**) or ALT (arrows) (**f**)

Next, telomere lengths were evaluated in the ALT-negative tumors, which was possible in 30 cases. Representative images of telomere lengths in NF1-associated gliomas are illustrated (Fig. 1a-c). Telomere lengths were normal in 17 (57%) cases and abnormally long in 12 (40%). Thus, ALT was a feature of predominantly high-grade or non-PA NF1-associated gliomas, while abnormally long telomeres (in the absence of ALT) are present in a subset of low-grade astrocytomas.

### ALT and short telomere lengths are limited to malignant subtypes among NF1-associated nerve sheath tumors

Next, we studied 197 samples obtained from 174 MPNSTs from 152 patients (93 NF1-associated, 16 non-syndrome-associated, 1 Schwannomatosis-associated, and 42 with unknown syndrome status given incomplete clinical history). Clinicopathologic and molecular features are outlined for all nerve sheath tumors (Additional file 1: Table S1) and summarized for the MPNST and neurofibroma cohorts in Table 1. ALT was present in 25 (of 148; 17%) MPNSTs from unique patients. In contrast, ALT was present in only 2 (of 193) neurofibromas obtained from 77 NF1 patients, a statistically significant difference (*p* < 0.0001, Fisher Exact Test). Notably, the 2 ALT-positive neurofibromas were obtained from a single patient (case 78) who underwent malignant transformation into an ALT-positive MPNST (Additional file 1: Figure S1). Tumors from this patient demonstrated partial ATRX and DAXX protein loss but lacked definite *ATRX* and *DAXX* mutations (Additional file 1: Table S1 and Table S2).

We assessed telomere lengths in a subset of ALT-negative MPNST cases (*n* = 30), identifying 14 (47%) cases with short, 15 (50%) cases with normal, and 1 (3%) case with long telomere lengths. Representative images of telomere lengths in MPNST are illustrated in Fig. 1d-f. When limiting to only the NF1-associated group, ALT was present in 9 (12%), while telomere lengths were abnormally short in 9 (of 24 ALT-negative cases; 38%), normal in 14 (58%), and long in a single case (4%). When limiting to only the sporadic group, ALT was present in 2 (of 14; 14%) MPNSTs, and telomere lengths were all short in the 5 evaluable ALT-negative cases. A total of 183 (of 191) ALT-negative neurofibromas were evaluable for telomere lengths and all cases displayed normal telomere lengths.

Within the ALT-positive MPNSTs, 6 (of 10) from distinct patients had ATRX protein loss, which was complete in 2 and partial in 4. Partial DAXX loss was present in 1 (of 10) cases. In this group, all 10 cases showed retained H3K27 trimethylation. In addition, one MPNST case displayed ATRX protein loss and was ALT-positive in the primary, and also displayed ATRX protein loss and was ALT-positive in 3 additional distant metastases, suggesting that ATRX loss and ALT are maintained during tumor progression. When assessing the ALT-negative cases, 11 (of 53; 21%) had partial ATRX loss and 3 (of 32; 9%) had partial DAXX loss. These findings collectively suggest that abnormal telomere lengths are frequent in MPNST, in contrast to their benign neurofibroma counterparts, and are characterized by the presence of ALT or abnormally shortened telomeres.

Next, we evaluated other rare NF1-associated solid tumors (*n* = 46). A single malignant phyllodes tumor of the breast demonstrated ALT; however, retained ATRX protein expression. In contrast, none of the other rare NF1-associated tumors demonstrated ALT (Additional file 1: Table S3), including gastrointestinal stromal tumors (GIST), pheochromocytomas, glomus tumors, juvenile xanthogranulomas, and duodenal neuroendocrine tumors.

### Genetic alterations associated with ALT in NF1-associated tumors

To study genetic alterations associated with the development of ALT, we performed NGS in 4 MPNSTs (3 ALT-positive and 1 ALT-negative) and 6 ALT-positive gliomas, 4 of which were also analyzed as part of a prior study [31]. Sequencing results of ALT-positive cases are provided by case in Additional file 1: Table S2. In the ALT-positive glioma group, all 6 had pathogenic *NF1* mutations, with a high VAF suggestive of LOH (*n* = 4) or a second mutation (*n* = 1). Three (of 6) had *ATRX* mutations, 2 had homozygous *CDKN2A/B* deletions, but none had a *DAXX* mutation.

**Fig. 2:**
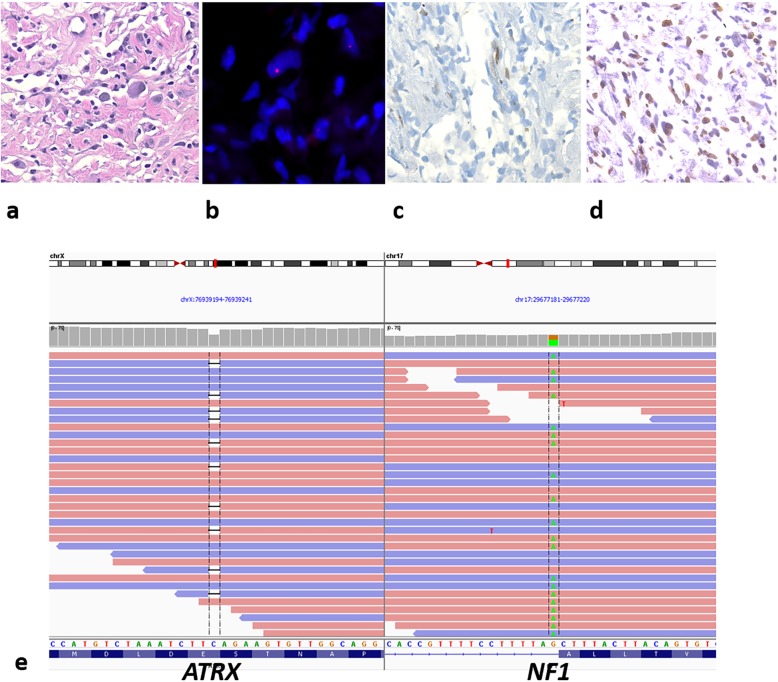
ALT-positive MPNST represent a distinct molecular subgroup. ALT-positive NF1-MPNST (case 114) (**a**), with ALT (**b**), ATRX protein loss (**c**), but preserved H3K27 trimethylation (**d**). Next generation sequencing demonstrated concurrent *ATRX*p.E511Kfs*3 and a *NF1* splice site mutation (c.7259-1G > A) (**e**), but no *EED* or *SUZ12* mutations

All 3 ALT-positive MPNST had pathogenic *NF1* mutations and lacked *EED*, *SUZ12,* or *DAXX* mutations. Case 110 had an *ATRX* p.E511Kfs*3 mutation with ATRX protein loss (Fig. 2). Case 78 had an *ATRX* variant (p.Q929E) with a 67% VAF and partial ATRX protein loss; whereas case 53 lacked an *ATRX* mutation, but contained two *RECQL4* variants (Additional file 1: Table S2). RECQL4 is a DNA helicase and alterations in genes associated with DNA repair could lead to ALT, in the absence of the better known alterations in the chromatin remodelers ATRX and DAXX. *RECQL4* variants were also present in two ALT-positive, ATRX intact NF1-gliomas. The ALT-negative MPNST with sequencing data displayed *NF1* and *SUZ12* mutations. Next, we analyzed publicly available gene sequencing data from cBioPortal [6, 11] and identified missense *DAXX* mutations in 2 (of 15) MPNSTs, both NF1-associated. These tumors lacked PRC2 (*EED*, *SUZ12*) mutations, and no mutations were present in *RBL2* or *SP100*, two altered telomere maintenance genes found to be enriched in ALT-positive leiomyosarcomas [7]. Collectively, these findings suggest that in a subset of NF1-associated tumors, ALT may be mediated by genes other than *ATRX* and *DAXX*.

### ALT is associated with shorter overall survival in NF1-associated glioma

**Fig. 3:**
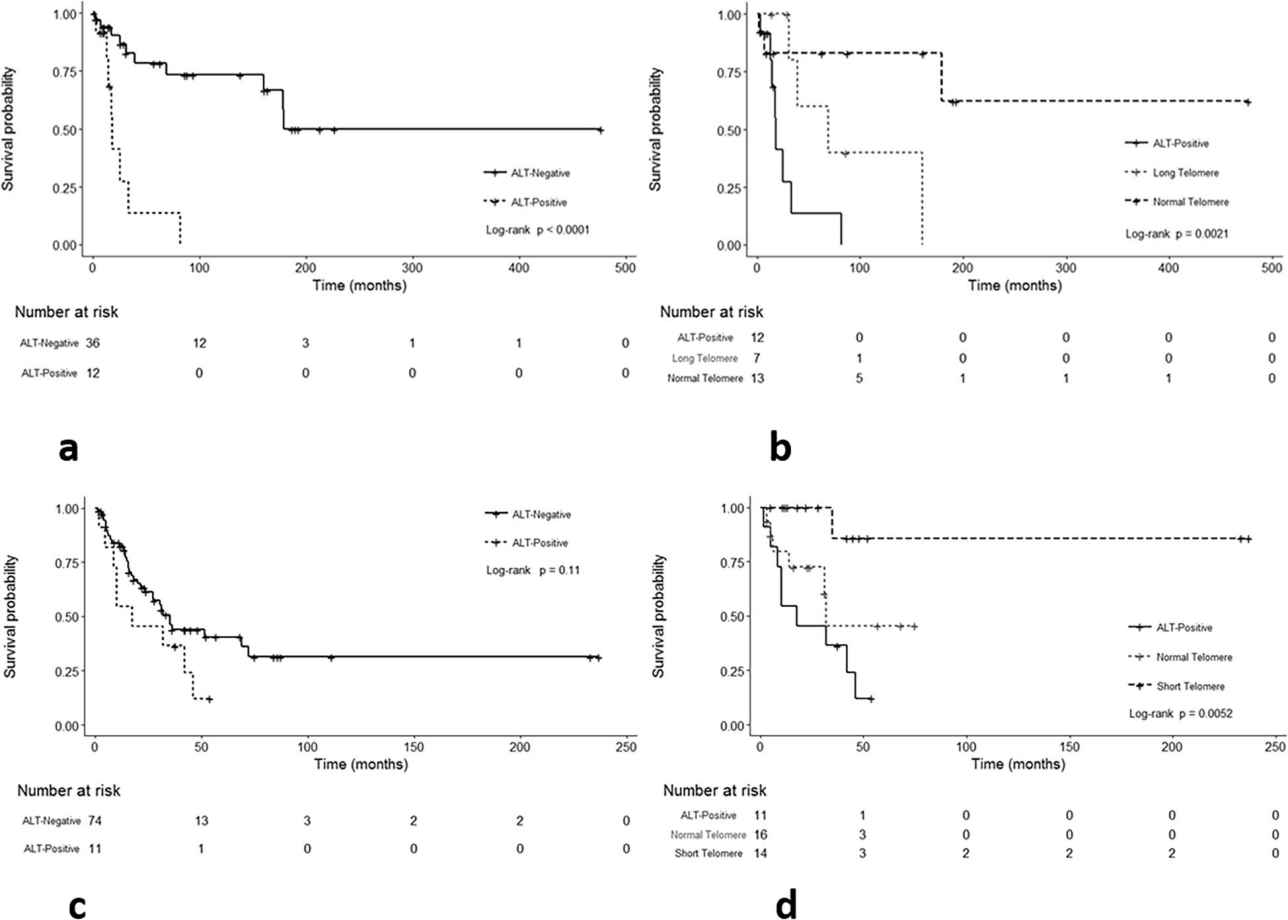
Telomere alterations are associated with overall survival in NF1-associated glioma and MPNST. Kaplan-Meier curves and log rank tests demonstrate worse overall survival in patients with ALT-positive NF1-gliomas compared to patients with ALT-negative NF1-gliomas (*p* < 0.0001) (**a**). Additionally, overall survival was intermediate for patients with tumors with long telomeres (but lacking ALT), and longer for patients with tumors with normal telomeres (*p* = 0.0021) (**b**). In the MPNST cohort known to be NF1-associated, the survival difference between ALT-positive and ALT-negative tumors was not statistically significant (*p* = 0.11) (**c**). However, overall survival was inferior for tumors with ALT, intermediate for tumors with normal telomeres, and superior for tumors with short telomeres (*p* = 0.0052), when further subdivided (**d**)

In the NF1-glioma cohort, median overall survival for patients with an ALT-positive tumor was only 18 months and significantly differed compared to patients with either long (69 months) or normal telomeres (not reached) (*p* < 0.0001, log rank test). When restricting the analysis to cases with evaluable telomere lengths, there was also a trend for intermediate survival for patients with long telomeres and better survival for patients with normal telomeres, although the differences in survival appeared to be driven mostly by the ALT-positive group (*p* = 0.0021, log rank test) (Fig. 3a, b). When modeling in a Cox univariate model, we observed consistent patterns for altered telomeres lengths (Long: HR = 3.99, 95% CI = 0.70–22.77; ALT HR = 13.05, 95% CI = 2.35–72.43) when compared to normal telomere lengths (Additional file 1: Table S4).

Since age and grade are some of the most important prognostic factors for central nervous system tumors, and ALT was associated with adults and higher grade tumors in our prior study [39], we performed multivariate analyses adjusting for these two factors. While there was not a statistically significant difference by telomere length group (Additional file 1: Table S4), ALT status remained significantly associated with a worse outcome in the model (HR = 3.62, 95% CI 1.17–11.17; *p* = 0.03) (Table 2).

**Table 2:** Association of ALT status with overall survival in patients with glioma

			Univariate			Multivariate		
	Death/Censor	Person-time (months)	HR	(95% CI)	*P*-value	HR	(95% CI)	P-value
Age	18/30	3469	1.05	(1.02–1.08)	0.001	1.02	(0.98–1.05)	0.3
Grade								
Low	6/25	2762	1.00	(Ref)	–	1.00	(Ref)	–
High	12/5	707	6.69	(2.30–19.41)	0.0005	3.36	(0.94–12.10)	0.06
ALT status								
Negative	10/26	3221	1.00	(Ref)	–	1.00	(Ref)	–
Positive	8/4	248	7.86	(2.68–23.06)	0.0002	3.62	(1.17–11.17)	0.03

### Shorter telomeres are associated with better overall survival in NF1-associated MPNST

In the NF1-MPNST cohort, overall survival was decreased for patients with ALT-positive tumors, intermediate for those with normal telomeres, and increased for those with short telomeres (*p* = 0.0052). When evaluating the entire MPNST cohort (including sporadic tumors and tumors with unknown NF1-status), median survival for patients was 28 months for ALT-positive tumors, 31 months for ALT-negative tumors, 32 months for normal telomeres, and not reached for short telomeres (Additional file 1: Figure S2a,b). These survival differences were similar in the NF1-MPNST group (*p* = 0.0016) (Fig. 3c,d).

In contrast to the glioma cohort, there were no statistical significant differences in overall survival between ALT-positive and ALT-negative NF1-MPNSTs (*p* = 0.20). Similarly, using a Cox univariate model, the altered telomere length groups did not differ (Short HR = 0.13, 95% CI = 0.02–1.12; ALT HR = 1.93, 95% CI = 0.68–5.49) when compared to the normal telomere group. However, in a multivariate model with age and grade, the short telomere group displayed a significantly better overall survival difference (HR = 0.03, 95% CI = 0.01–0.29; *p* = 0.003) compared to the normal telomere group (Table 3). When considering only ALT status, there were no significant differences with ALT-positivity in the NF1-MPNST cohort (HR = 1.21, 95% CI = 0.41–3.59; *p* = 0.70) (Table 3) or in the entire MPNST group (including sporadic and unknown status) (Additional file 1: Table S5 and Table S6).

**Table 3:** Association of telomere lengths with overall survival in patients with NF1-associated MPNST

			Univariate			Multivariate		
	Death/Censor	Person-time (months)	HR	(95% CI)	P-value	HR	(95% CI)	P-value
Age	16/25	1482	1.01	(0.98–1.04)	0.5	1.08	(1.02–1.14)	0.006
Grade								
Low	1/3	168	1.00	(Ref)	–	1.00	(Ref)	–
High	15/22	1315	2.29	(0.30–17.52)	0.3	12.33	(0.42–28.97)	0.03
Telomere lengths								
Normal	6/10	417	1.00	(Ref)	–	1.00	(Ref)	–
Short	1/13	801	0.13	(0.02–1.12)	0.06	0.03	(0.01–0.29)	0.003
ALT	9/2	264	1.93	(0.68–5.49)	0.2	1.21	(0.41–3.59)	0.7

## Discussion

Identifying the genetic drivers and the precise sequence of genetic hits in NF1-associated tumor development is still being fully elucidated. However, we have previously demonstrated that functional loss of ATRX and activation of ALT are frequent features of diffuse and high-grade astrocytomas that develop in patients with NF1, and are present in 7 (58%) and 8 (67%) respectively [39]. More recently, D’angelo et al. identified *ATRX* mutations in 38% of NF1-associated high grade gliomas, compared to 3.1% of low-grade gliomas [8]. In addition, another group of aggressive gliomas characterized by a high frequency of ATRX alterations and ALT activation is PA with anaplasia, approximately one third of which are NF1-associated or demonstrate somatic *NF1* gene mutations [35, 37]. One important finding in our current study is that in NF1-associated gliomas, ALT is independently associated with worse survival in a multivariate model also accounting for grade and age, two key factors consistently associated with survival in gliomas.

ALT was also identified in one additional tumor type in our study (i.e. MPNST) albeit at a lower frequency (17%) compared to the gliomas. Only one patient had two ALT-positive neurofibromas in our study, and they were presumably precursors to a fatal ALT-positive MPNST. ALT was also identified in a single malignant phyllodes tumor, although ALT was uniformly absent in the remaining NF1-associated tumors (e.g. GIST, neuroendocrine tumors, and glomus tumors), suggesting that ALT in NF1-associated tumors is associated with a malignant phenotype. Our results are in agreement with the finding of ALT in 3 (of 14; 21%) MPNST in a prior study, only one of which had ATRX loss [22]. In addition, an *ATRX* variant was found in 1 (of 7) NF1-associated MPNSTs through next generation sequencing in a previous study [15]. More recently, Lu et al. reported aberrant ATRX immunoreactivity in 65% of NF1-associated MPNST, a finding associated with shorter overall survival [24]. Of note, in cBioPortal, *ATRX* mutations were present in 4 (2.5%) of 162 pheochromocytomas/ paragangliomas, another tumor type that develops in NF1 patients. Others have reported *ATRX* mutations in 12.6% of pheochromocytomas/paragangliomas, mostly associated with SDH alterations, but also in one tumor with an *NF1* mutation [10]. Additionally, telomerase activation and *ATRX* mutations were found to be independent factors for poor prognosis in pheochromocytomas/paragangliomas in a recent study [17]. Loss of ATRX expression and ALT are very rare in GIST in general, which is in accordance with our findings [1, 22]. However, we did not identify ATRX alterations in one of the 3 ALT-positive MPNST sequenced. We recently studied a group of low-grade gliomas developing in NF1 patients (“SEGA-like astrocytomas”) [16]. We have included these cases as part of this broader study, and found ALT was present in 4 (of 10; 40%) of these tumors. However, only one of these ALT-positive tumors contained a pathogenic *ATRX* mutation. These findings suggest that ALT may develop independently of ATRX and DAXX in subsets of NF1-associated tumors.

Another key finding in our study is that short telomeres were prevalent in ALT-negative MPNST, while long telomeres were present in ALT-negative gliomas, independent of grade. Telomere lengths in cancer cells can vary among different tumor types. For example, a systematic analysis of telomere lengths using sequencing data found that in most tumors telomeres were shorter than in normal tissues; however, longer telomeres were observed in gliomas and sarcomas [2]. These differences potentially reflect different age distributions, variation of telomere lengths among cell lineages, or may even reflect differences in the degree and timing of telomerase activation.

**Fig. 4:**
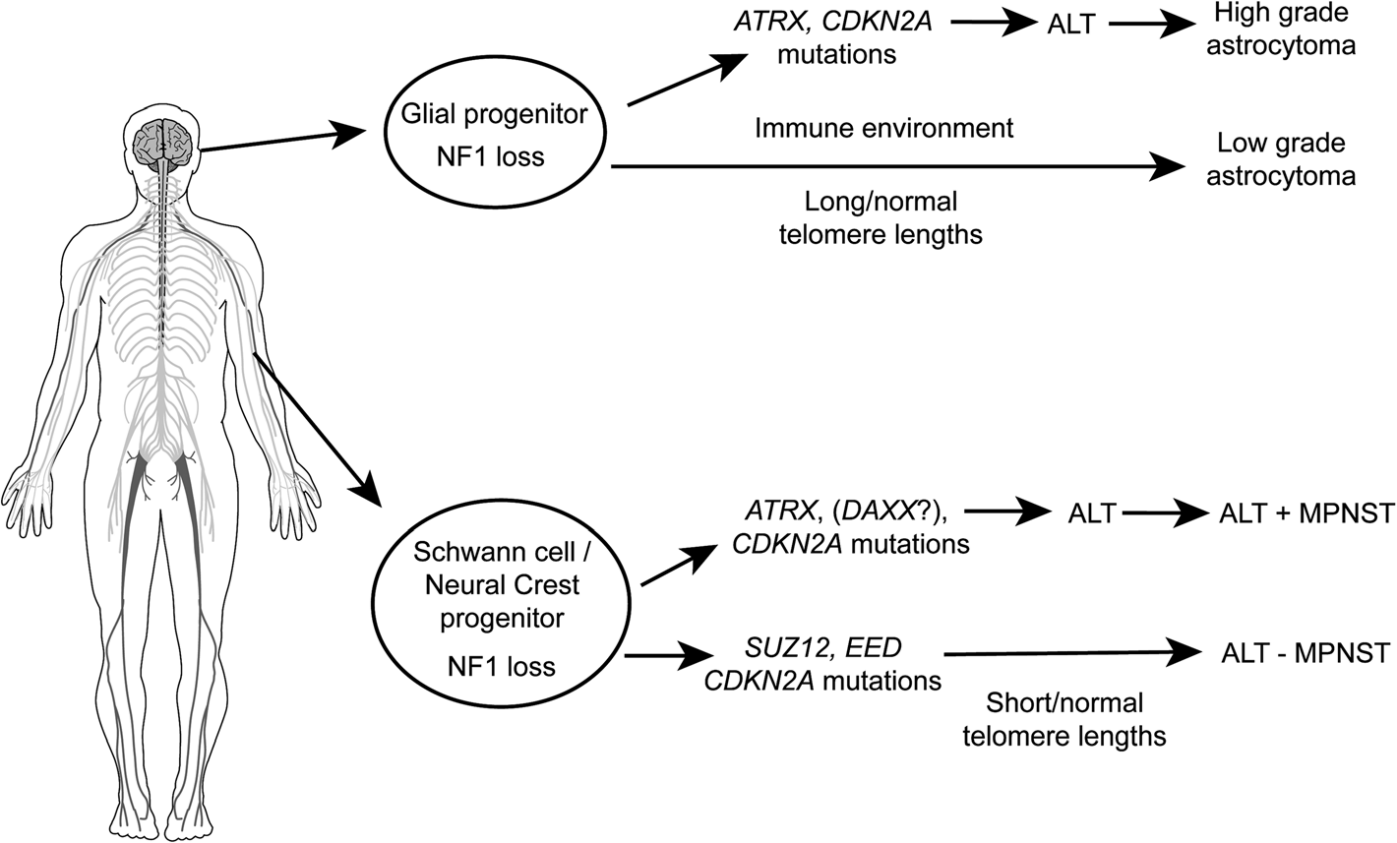
Telomere alterations and contribution to NF1 tumorigenesis. In gliomagenesis, ATRX alterations and ALT are associated with high grade tumors, while longer telomeres (without ALT) are frequent in both high grade and low grade gliomas. In malignant nerve sheath tumors, there are two separate pathways, one that involves ALT (with or without *ATRX* alterations), and another that lacks ALT and *ATRX* alterations, but frequently has *SUZ12* or *EED* alterations, loss of H3 K27 trimethylation and abnormally short telomeres. Some alterations are common to all malignant NF1-associated tumors, particularly *CDKN2A* deletions/mutations

Recent studies in MPNST have demonstrated frequent mutations in *SUZ12* and *EED* which encode protein components of the PRC2 complex [21, 43]. These alterations are associated with loss of H3K27me3 [32, 33, 40]. However, loss of H3K27me3 was not a feature in MPNST with ALT, and we did not identify *SUZ12* or *EED* mutations. These findings suggest that ALT-positive MPNST represents a distinctive molecular subgroup that may benefit from different therapeutic approaches (Fig. 4).

Prior studies suggest that *ATRX* mutation alone is not sufficient for tumorigenesis or the development of ALT. Rather, *ATRX* mutations in CNS tumors seem to develop in the context of other more basic genetic drivers to facilitate tumorigenesis. In the context of NF1-associated tumors, this seems to be *NF1* loss, while IDH mutations and histone H3 mutations (particularly G34) frequently coexist with ATRX loss in other tumor subsets [5, 20, 42]. In the rare phenomenon of pilocytic astrocytomas with anaplasia, ATRX loss frequently co-occurs with NF1 loss, as well as mutually exclusive alterations in the members of the MAPK pathway (e.g. *BRAF)* [35, 37]. Detecting ATRX alterations may also have management implications for NF1 patients with gliomas, since when present they identify subgroups that are clinically more aggressive. ATRX loss is routinely tested for in the laboratory setting through immunohistochemistry or sequencing. In contrast, while ALT is not tested for clinically, it has the potential to be used in cases with equivocal ATRX findings, either as by FISH or CISH.

One of the limitations of our studies includes that we did not find a specific explanation for the development of ALT in a subset of cases lacking *ATRX* or *DAXX* mutations. The NGS panel applied covers many genes, but it could have missed other ALT related genes that in the future may be identified by more comprehensive (whole exome/genome) sequencing methods. Additionally, the number of rare NF1-associated solid tumors in our study is relatively low. Although no clear patterns for telomere alterations were noted, future studies with larger number of cases may be required for confirmation of our findings.

In summary, our data support an important role for ATRX/DAXX loss and acquisition of ALT with an aggressive biology in NF1-associated gliomas, as well as a molecular subset of NF1-associated MPNSTs.

## Additional file

Additional file 1: **Figure S1.** MPNST case 78. **Figure S2.** Telomere alterations are associated with overall survival in combined MPNST group. **Table S1.** Clinicopathologic Features of NF1-Associated Solid Tumors and MPNST (337 patients). **Table S2.** Next generation sequencing results of ALT positive NF1-associated gliomas and MPNST (*n* = 9). **Table S3.** Telomere alterations in rare NF1-associated tumors (*n* = 46). **Table S4.** Association of telomere length with overall survival in patients with glioma. **Table S5**. Association of telomere lengths with overall survival in all patients with MPNST. **Table S6**. Association of ALT with overall survival in all patients with MPNST. (PDF 3427 kb)
