# Supplementary material for: Telomere alterations in neurofibromatosis type 1-associated solid tumors
Source: Acta Neuropathol Commun. 2019 Aug 28;7:139. doi: 10.1186/s40478-019-0792-5 (PMC6712691; doi:10.1186/s40478-019-0792-5)
Supplement: Supplementary file 1 — Figure S1. MPNST case 78. Figure S2. Telomere alterations are associated with overall survival in combined MPNST group. Table S1. Clinicopathologic Features of NF1-Associated Solid Tumors and MPNST (337 patients). Table S2. Next generation sequencing results of ALT positive NF1-associated gliomas and MPNST (n = 9). Table S3. Telomere alterations in rare NF1-associated tumors (n = 46). Table S4. Association of telomere length with overall survival in patients with glioma. Table S5. Association of telomere lengths with overall survival in all patients with MPNST. Table S6. Association of ALT with overall survival in all patients with MPNST. (PDF 3427 kb) [file 40478_2019_792_MOESM1_ESM.pdf]

Telomere alterations in neurofibromatosis type 1-associated solid tumors

Fausto J. Rodriguez[1,5]*, Mindy K. Graham[1], Jacqueline A. Brosnan-Cashman[1], John R. Barber[6] Christine Davis[1], M. Adelita Vizcaino[1], Doreen N. Palsgrove[1,5], Caterina Giannini[7], Melike Pekmezci[8], Sonika Dahiya[9], Murat Gokden[10], Michael Noë[1], Laura D. Wood[1,5], Christine A. Pratilas[5], Carol Morris[4], Allan Belzberg[3], Jaishri Blakeley[2,5], Christopher M. Heaphy[1,5]

Departments of Pathology[1], Neurology[2] , Neurosurgery[3], Orthopedics[4], and Sidney Kimmel Comprehensive Cancer Center[5], Johns Hopkins University School of Medicine, Baltimore, MD; Department of Epidemiology, Johns Hopkins Bloomberg School of Public Health[6], Baltimore, MD; Department of Laboratory Medicine and Pathology, Mayo Clinic[7], Rochester, MN; Department of Pathology, University of California San Francisco[8], San Francisco, CA; Department of Pathology, Washington University[10], St. Louis, MO; University of Arkansas[10], Little Rock, AR

*To whom correspondence should be addressed. E-mail: frodrig4@jhmi.edu

This PDF includes:
Figures S1-S2
Tables S1-S6

Figure S1. MPNST case 78. Over a number of decades this ALT positive low grade MPNST with ALT but only partial ATRX evolved from a diffuse/cellular neurofibroma lacking ALT (left column) to a neurofibroma with atypia and ALT (middle two columns) all the way to ALT positive MPNST (arrows denote large telomeric foci on telomere FISH indicative of ALT).

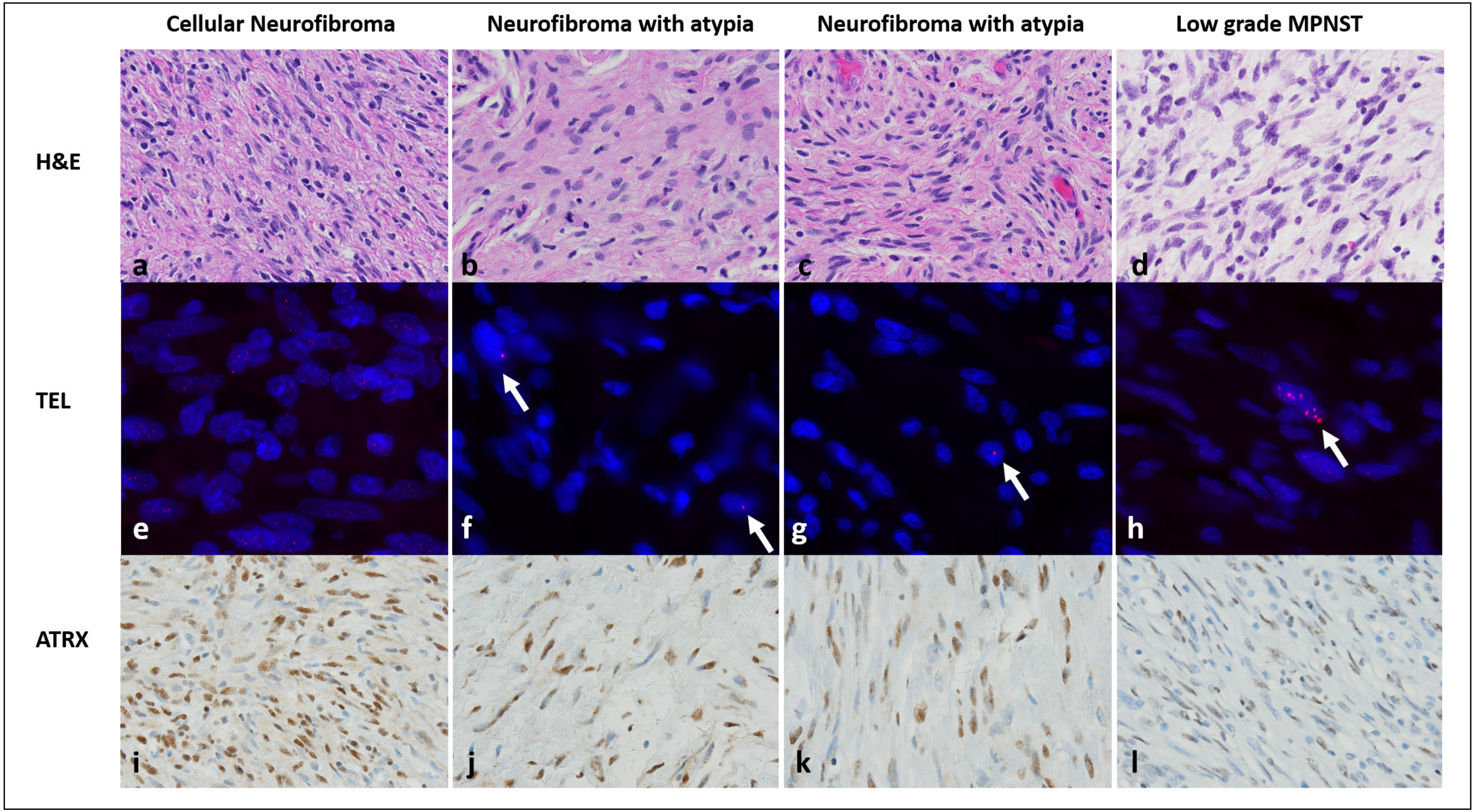

Figure S2. Telomere alterations are associated with overall survival in combined MPNST group. In the MPNST cohort, the survival difference between ALT-positive and ALT-negative tumors was not statistically significant (p=0.14) (**C**). However, overall survival was inferior for tumors with ALT, intermediate for tumors with normal telomeres, and superior for tumors with short telomeres (p=0.0016), when further subdivided (**D**).

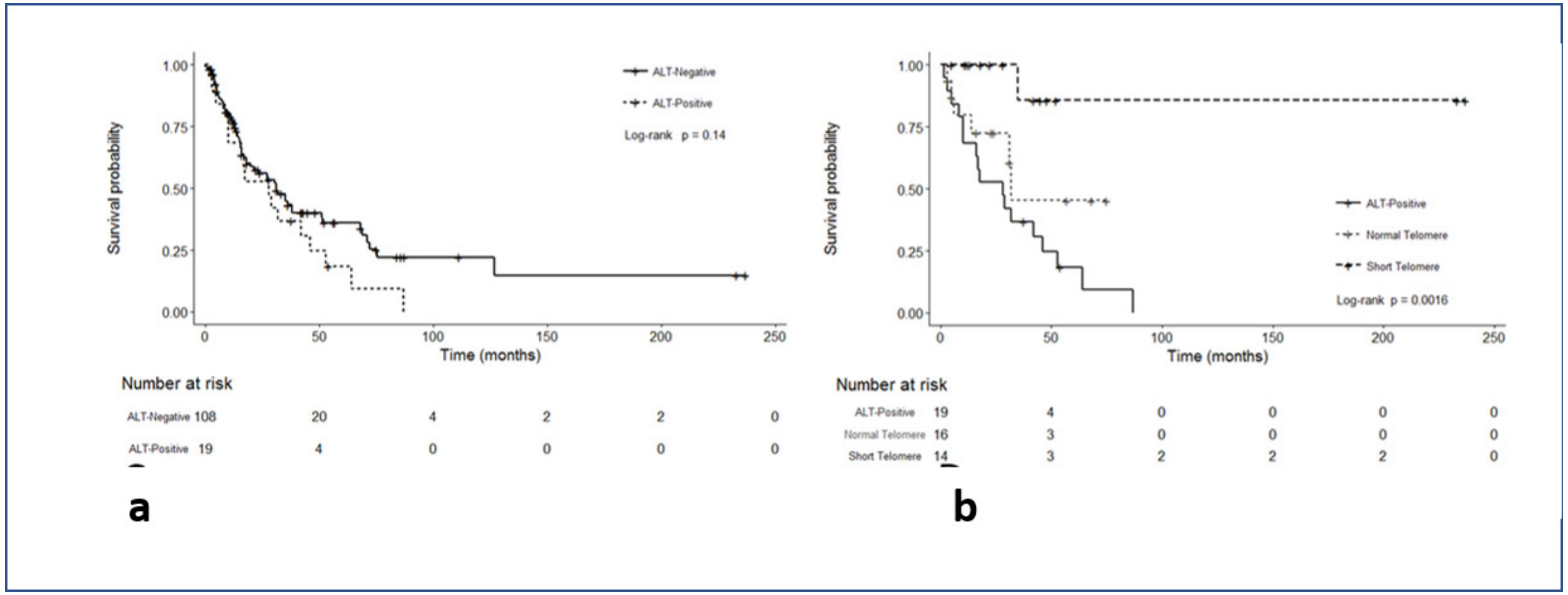

Table S1. Clinicopathologic Features of NF1-Associated Solid Tumors and MPNST (337 patients)

PT #	Age at Dx (years)	Sex	Tumor Location	Specific Diagnosis	Grade	NF1 Status	ALT	Telomere Lengths	ATRX IHC	DAXX IHC	Individual distinct patient =1	Individual distinct tumor=1	Primary=1, recurrence=2, metastasis=3
**1**	52	M	R periocular	Neurofibroma	Benign	NF1	NEGATIVE	Normal			1	1	1
**2**	18	M	L eye	Neurofibroma	Benign	NF1	NEGATIVE	Normal	Preserved		1	1	1
**3**	41	M	Thoracic spine	MPNST	High grade	NF1	NEGATIVE	Normal	Partial loss		1	1	1
**4**	38	M	L neck	Cutaneous neurofibroma	Benign	NF1	NEGATIVE	Normal	Preserved		1	1	1
4	38		L nasal	Cutaneous neurofibroma	Benign	NF1	NEGATIVE	Normal	Preserved			1	1
4	38		Forehead	Cutaneous neurofibroma	Benign	NF1	NEGATIVE	Normal	Preserved			1	1
4	38		L canthus	Cutaneous neurofibroma	Benign	NF1	NEGATIVE	Normal	Preserved			1	1
**5**	7	F	Thalamus	Pilocytic astrocytoma	Low grade	NF1	NEGATIVE		Preserved	Preserved	1	1	2
**6**	38	M	R sciatic nerve	MPNST	High grade	NF1	NEGATIVE	Short	Preserved		1	1	1
**7**	9	M	Cerebellum	Pilocytic astrocytoma	Low grade	NF1	NEGATIVE	Normal			1	1	1
**8**	31	F	skin	Neurofibroma	Benign	NF1	NEGATIVE	Normal	Preserved		1	1	1
**9**	13	F	Pelvis	MPNST	High grade	NF1	NEGATIVE	Short	Preserved		1	1	1
**10**	20	F	Temporal lobe	Gliosarcoma	High grade	NF1	NEGATIVE	Normal	Preserved	Preserved	1	1	1
10	18	F	Mediatinum	MPNST	High grade	NF1	NEGATIVE		Preserved			1	1
10	18	F	Mediatinum	Neurofibroma	Benign	NF1	NEGATIVE					1	1
**11**	37	F	Frontal lobe	Anaplastic astrocytoma	High grade	NF1	POSITIVE	ALT	Loss	Preserved	1	1	1
11	42	F	Frontal lobe	Anaplastic astrocytoma	High grade	NF1	POSITIVE	ALT	Loss	Preserved			2
12				Angiolipoma	Benign	NF1	NEGATIVE	Normal				1	1
12				Angiolipoma	Benign	NF1	NEGATIVE	Normal				1	1
12				Lipoma	Benign	NF1	NEGATIVE	Normal				1	1
12				Lipoma	Benign	NF1	NEGATIVE	Normal				1	1
**12**				Lipoma	Benign	NF1	NEGATIVE				1	1	1
12				Lipoma	Benign	NF1						1	1
12				Plexiform neurofibroma	Benign	NF1	NEGATIVE	Normal				1	1
**13**	27	M	Brainstem	Anaplastic astrocytoma	High grade	NF1	POSITIVE	ALT	Loss		1	1	1
**14**	33	M	Pleura	MPNST	High grade	NF1	NEGATIVE	Normal	Preserved		1	1	2
**15**	5	M	R orbit	Plexiform neurofibroma	Benign	NF1	NEGATIVE	Normal	Preserved		1	1	1
**16**	76	F	Sciatic nerve	MPNST	High grade	sporadic	NEGATIVE	Short			1	1	1
**17**	19	M	skin	Plexiform neurofibroma	Benign	NF1	NEGATIVE	Normal	Partial loss		1	1	1
**18**	31	M	L orbit	Plexiform neurofibroma	Benign	NF1	NEGATIVE	Normal	Preserved		1	1	2
**19**	27	M	L axilla	Neurofibroma	Benign	NF1	NEGATIVE	Normal	Preserved		1		2

19	19	M	Brachial plexus	Neurofibroma	Benign	NF1	NEGATIVE	Normal	Preserved			1	1
**20**	33	M	L sciatic/femoral nerve	Plexiform neurofibroma	Benign	NF1	NEGATIVE	Normal	Preserved		1	1	1
**21**	17	F	Spine	Atypical neurofibroma	Benign	NF1	NEGATIVE		Preserved		1	1	1
21	17	F	L leg	MPNST	High grade	NF1	NEGATIVE	Short	Preserved			1	1
**22**	37	F	R adrenal gland	Pheochromocytoma	Benign	NF1	NEGATIVE	Short	Preserved		1	1	1
22	37	F	Pericaval	Plexiform neurofibroma	Benign	NF1	NEGATIVE	Normal	Preserved			1	1
22	37	F	Duodenum-medial	GIST	Low grade	NF1	NEGATIVE	Normal	Preserved			1	1
22	37	F	Duodenum-lateral	GIST	Low grade	NF1	NEGATIVE	Normal	Preserved			1	1
**23**	20	M	L thigh	Glomangioma	Benign	sporadic	NEGATIVE	Normal	Preserved		1	1	1
**24**	13	F	Brainstem	Pilocytic astrocytoma	Low grade	NF1			Preserved		1	1	1
**25**	32	F	Cerebellum	Pilocytic astrocytoma	Low grade	NF1	POSITIVE	ALT	Loss		1	1	1
**26**	14	F	Thalamus	Pilocytic astrocytoma	Low grade	NF1	NEGATIVE		Partial loss	Preserved	1	1	1
**27**	9	M	Spinal cord	Glioblastoma	High grade	NF1	NEGATIVE		Preserved	Preserved	1	1	1
**28**	34	M	Femur	MPNST	High grade	sporadic	NEGATIVE	Short	Preserved		1	1	1
29	52	M	L scalp	Diffuse neurofibroma	Benign	NF1	NEGATIVE	Normal	Preserved			1	1
**29**	52	M	L adrenal gland	Pheochromocytoma	Benign	NF1	NEGATIVE				1	1	1
**30**	12	M	retroperitoneum	MPNST	High grade	NF1	NEGATIVE	Normal	Preserved		1	1	1
30	12	M		Plexiform neurofibroma	Benign	NF1	NEGATIVE	Normal	Preserved			1	1
**31**	34	M	cerebellum	Diffuse Astrocytoma	Low grade	NF1	NEGATIVE	Long	Preserved		1	1	1
**32**	38	F	skin	Neurofibroma	Benign	NF1	NEGATIVE	Normal	Preserved		1	1	1
**33**	46	M	skin	Neurofibroma	Benign	NF1	NEGATIVE	Normal	Partial loss		1	1	1
33	55	M	skin	Neurofibroma	Benign	NF1	NEGATIVE	Normal	Preserved			1	1
33	46	M		Plexiform neurofibroma	Benign	NF1	NEGATIVE					1	1
**34**	52	M	L brow	Neurofibroma	Benign	NF1	NEGATIVE	Normal	Preserved		1	1	1
**35**	8	M	Spine	Atypical neurofibroma	Benign	NF1	NEGATIVE	Normal	Preserved		1	1	1
**36**	59	F	R thumb	Glomus tumor	Benign	NF1	NEGATIVE	Normal			1	1	1
36	59	F	skin	Neurofibroma	Benign	NF1	NEGATIVE	Normal				1	1
36	59	F	skin	Neurofibroma	Benign	NF1	NEGATIVE	Normal				1	1
36	59	F	R small finger	Plexiform neurofibroma	Benign	NF1	NEGATIVE	Normal				1	1
**37**	25	M	radial nerve	MPNST	High grade	NF1	NEGATIVE	Short	Preserved		1	1	1
**38**	11	M	Occipital lobe	Low grade astrocytoma	Low grade	NF1	NEGATIVE		Preserved	Preserved	1	1	1
**39**	13	M	4th ventricle	Pilocytic astrocytoma	Low grade	NF1	NEGATIVE		Preserved	Preserved	1	1	1
**40**	18	M	R orbit	Neurofibroma	Benign	NF1	NEGATIVE	Normal			1	1	1
**41**	7	M	Forehead	Plexiform neurofibroma	Benign	NF1	NEGATIVE	Normal	Preserved		1	1	1
**42**	45	M	L calf	Dermatofibroma	Benign	NF1	NEGATIVE	Normal			1	1	1
43	60	F	L small finger	Glomus tumor	Benign	NF1	NEGATIVE	Normal	Preserved			1	1

**43**	60	F	L index finger	Neurofibroma	Benign	NF1	NEGATIVE	Normal	Preserved		1	1	1
43	60	F	L small finger	Neurofibroma	Benign	NF1	NEGATIVE	Normal	Preserved			1	1
44	44	F	Calf	Massive soft tissue neurofibroma	Benign	NF1	NEGATIVE	Normal	Preserved			1	1
**44**	44	F	scalp	Neurofibroma	Benign	NF1	NEGATIVE	Normal	Preserved		1	1	1
**45**	20	M	R paraspinal	Neurofibroma	Benign	NF1	NEGATIVE	Normal	Preserved		1	1	1
45	21	M	L Paraspinal	Neurofibroma	Benign	NF1			Preserved			1	1
45	21	M	R thigh	Neurofibroma	Benign	NF1			Preserved			1	1
45	18	M	L neck	Syringocystadenoma papilliferum	Benign	NF1	NEGATIVE	Normal	Preserved			1	1
46	50	F	ampulla	Gangliocytic paraganglioma	Benign	NF1	NEGATIVE	Normal					1
**46**	50	F	Ampulla	Gangliocytic paraganglioma	Benign	NF1	NEGATIVE				1	1	1
47	37	F	soft tissue	Plexiform neurofibroma	Benign	NF1	NEGATIVE		Partial loss			1	1
**47**	37	F	skin	Neurofibroma	Benign	NF1	NEGATIVE	Normal	Preserved		1	1	1
47	37	F	soft tissue	Plexiform neurofibroma	Benign	NF1			Preserved			1	1
48	34	F	L orbit	Neurofibroma	Benign	NF1	NEGATIVE	Normal	Preserved			1	1
**48**	23	F	R shoulder	Plexiform neurofibroma	Benign	NF1	NEGATIVE	Normal	Preserved		1	1	1
48	23	F	back	Plexiform neurofibroma	Benign	NF1	NEGATIVE	Normal	Preserved			1	1
48	23	F	L neck	Plexiform neurofibroma	Benign	NF1	NEGATIVE	Normal				1	1
**49**	31	F	R foot	Atypical neurofibroma	Benign	Schwannomatosis	NEGATIVE	Normal	Preserved		1	1	1
**50**	25	F	Groin	Diffuse neurofibroma	Benign	NF1	NEGATIVE	Normal	Preserved		1	1	1
50	25	F	L breast	Neurofibroma	Benign	NF1	NEGATIVE	Normal	Preserved			1	1
50	25	F	L neck	Neurofibroma	Benign	NF1	NEGATIVE	Normal	Preserved			1	1
**51**	4	M	Suprasellar region	Pilocytic astrocytoma	Low grade	NF1	NEGATIVE		Preserved		1	1	2
51	17	M	Parietal lobe	Pilocytic astrocytoma	Low grade	NF1	NEGATIVE		Preserved	Preserved		1	1
**52**	41	M	R neck	MPNST	High grade	NF1	NEGATIVE	Short	Preserved	Partial loss	1	1	2
**53**	60	F	L knee	MPNST	High grade	NF1	POSITIVE	ALT	Partial loss	Preserved	1	1	1
53	49	F	L upper lid	Diffuse neurofibroma	Benign	NF1	NEGATIVE	Normal	Preserved			1	1
**54**	42	F	L buttock	Massive soft tissue neurofibroma	Benign	NF1	NEGATIVE	Normal	Preserved		1	1	1
**55**	11	M	Spinal cord	Diffuse Astrocytoma	Low grade	NF1	NEGATIVE		Preserved	Preserved	1	1	1
**56**	20	M	Jejunum	GIST	Low grade	NF1	NEGATIVE	Short			1	1	1
**57**	15	F	scalp	Plexiform neurofibroma	Benign	NF1	NEGATIVE	Normal	Preserved		1	1	1
**58**	26	F	Proximal femur	MPNST	High grade	NF1	NEGATIVE	Normal	Preserved		1	1	3
**59**	15	M	R frontal lobe	Low grade astrocytoma	Low grade	NF1	POSITIVE	ALT	Preserved	Preserved	1	1	1
**60**	20	F	L frontal lobe	SEGA-like astrocytoma	Low grade	NF1	POSITIVE	ALT	Preserved	Preserved	1	1	1
60	21	F	chest wall	Cutaneous neurofibroma	Benign	NF1	NEGATIVE	Normal	Preserved			1	1
60	21	F	Left arm	Cutaneous neurofibroma	Benign	NF1	NEGATIVE	Normal	Preserved			1	1
60	21	F	Left hip	Diffuse neurofibroma	Benign	NF1	NEGATIVE	Normal	Preserved			1	1

**61**	28	M	Mediastinum	MPNST	High grade	NF1	NEGATIVE	Normal	Partial loss	Preserved	1	1	1
61	28	M	Mediastinum	MPNST	High grade	NF1	NEGATIVE	Short	Preserved	Preserved		0	1
61	28	M	R neck	ANNUBP	Benign	NF1	NEGATIVE	Normal	Preserved			1	1
61	28	M	L thigh	Neurofibroma	Benign	NF1	NEGATIVE	Normal	Preserved			1	1
**62**	61	M	R lower lid	Neurofibroma	Benign	NF1	NEGATIVE	Normal	Preserved		1	1	1
63	37	M	Axilla	Atypical neurofibroma	Benign	NF1	NEGATIVE		Partial loss			1	1
**63**	37	M	Lumbar-paraspinal	MPNST	Low grade	NF1	NEGATIVE	Normal	Preserved		1	1	2
64	64	F	gluteus	Atypical neurofibroma	Benign	NF1			Partial loss			1	1
**64**	64	F	R sciatic nerve	Atypical neurofibroma	Benign	NF1	NEGATIVE	Normal	Preserved		1	1	2
**65**	33	F	R paraspinal	MPNST	High grade	NF1	POSITIVE	ALT	Partial loss	Preserved	1	1	1
65	33	F	Lung	MPNST	High grade	NF1	POSITIVE	ALT	Partial loss	Preserved		1	3
65	33	F	lung	MPNST	High grade	NF1	POSITIVE	ALT	Partial loss	Preserved		1	3
65	34	F	lung	MPNST	High grade	NF1	POSITIVE	ALT	Preserved	Preserved		1	3
**66**	64	M	L brachial plexus	MPNST		sporadic	NEGATIVE	Short	Preserved		1	1	1
**67**	52	M	Femoral nerve	MPNST	High grade	NF1	NEGATIVE	Normal	Preserved		1	1	1
67	52	M	Femoral nerve	MPNST	High grade	NF1	NEGATIVE	Short				1	1
**68**	16	F	Frontal lobe	Oligodendroglioma, IDH mutant and 1p/19q codeleted	Low grade	NF1	NEGATIVE	Long	Preserved		1	1	1
**69**	36	F	R adrenal gland	Pheochromocytoma		NF1	NEGATIVE		Preserved		1	1	1
69	36	F	L adrenal gland	Pheochromocytoma		NF1	NEGATIVE		Preserved			1	1
**70**	25	M	R thalamus	Diffuse Astrocytoma	Low grade	NF1	POSITIVE	ALT	Loss	Preserved	1	1	1
70	26	M	R thalamus	Diffuse Astrocytoma	Low grade	NF1	POSITIVE	ALT					2
**71**	2	M	periorbital	Plexiform neurofibroma	Benign	NF1	NEGATIVE	Normal	Partial loss		1	1	2
**72**	50	M	L face	Spindle cell tumor	Low grade	NF1	NEGATIVE	Normal	Preserved		1	1	1
**73**	9	M	R orbit	Plexiform neurofibroma	Benign	NF1	NEGATIVE	Normal	Preserved		1	1	1
**74**	19	F	L orbit	Plexiform neurofibroma	Benign	NF1	NEGATIVE	Normal	Preserved		1	1	1
74	22	F	L orbit	Plexiform neurofibroma	Benign	NF1			Preserved				2
74	21	F	L eyelid	Diffuse neurofibroma	Benign	NF1	NEGATIVE	Normal					2
74	20	F	L orbit	Diffuse neurofibroma	Benign	NF1	NEGATIVE	Normal					2
**75**	9	F	Tectum	Low grade astrocytoma	Low grade	NF1	NEGATIVE		Preserved	Preserved	1	1	1
**76**	40	F	R orbit	Diffuse neurofibroma	Benign	NF1	NEGATIVE	Normal			1	1	1
77	35	F	R thigh	Localized intraneural neurofibroma	Benign	NF1	NEGATIVE	Normal	Preserved			1	1
77	35	F	R waist	Localized intraneural neurofibroma	Benign	NF1	NEGATIVE	Normal	Preserved			1	1
**77**	26	F	Sacrum	Neurofibroma	Benign	NF1	NEGATIVE	Normal	Preserved		1	1	1
77	35	F	L thigh	Plexiform neurofibroma	Benign	NF1	NEGATIVE	Normal	Preserved			1	1
**78**	62	F	Intracranial	MPNST	Low grade	NF1	POSITIVE	ALT	Partial loss	Partial loss	1	1	1
78	60	F	scalp	Atypical neurofibroma	Benign	NF1	POSITIVE	ALT	Partial loss	Preserved		1	2

78	60	F	R temporal	Diffuse neurofibroma	Benign	NF1	POSITIVE	ALT	Partial loss	Preserved			2
78	58	F	Back-skin	Cutaneous neurofibroma	Benign	NF1	NEGATIVE	Normal	Partial loss			1	1
78	58	F	Back-skin	Cutaneous neurofibroma	Benign	NF1	NEGATIVE	Normal	Partial loss			1	1
78	58	F	R buttock	Malignant spindle cell neoplasm	High grade	NF1	POSITIVE	ALT	Partial loss				3
78	58	F	F forehead	Diffuse neurofibroma	Benign	NF1	NEGATIVE	Normal	Partial loss				2
78	57	F	R orbit	Diffuse neurofibroma	Benign	NF1	NEGATIVE	Normal	Preserved				2
78	56	F	L small finger	Glomus tumor	Benign	NF1	NEGATIVE		Preserved			1	1
78	56	F	R eyelid	Neurofibroma	Benign	NF1	NEGATIVE	Normal	Preserved				2
78	56	F	L small finger	Traumatic neuroma	Benign	NF1	NEGATIVE	Normal	Preserved			1	1
78	58	F	Back-skin	Cutaneous neurofibroma	Benign	NF1	NEGATIVE	Normal				1	1
78	58	F	Back-skin	Cutaneous neurofibroma	Benign	NF1	NEGATIVE	Normal				1	1
78	58	F	Back-skin	Cutaneous neurofibroma	Benign	NF1	NEGATIVE	Normal				1	1
78	58	F	Back-skin	Cutaneous neurofibroma	Benign	NF1	NEGATIVE	Normal				1	1
78	55	F	R orbit	Diffuse neurofibroma	Benign	NF1	NEGATIVE	Normal					2
**79**	9	M	L eyelid	Plexiform neurofibroma	Benign	NF1	NEGATIVE	Normal	Preserved		1	1	1
80	30	M	R buttock	Diffuse neurofibroma	Benign	NF1	NEGATIVE	Normal	Preserved			1	1
**80**	30	M	R shoulder	Neurofibroma	Benign	NF1	NEGATIVE	Normal	Preserved		1	1	1
80	30	M	R elbow	Neurofibroma	Benign	NF1	NEGATIVE	Normal	Preserved			1	1
**81**	1	F	Eyelid	Plexiform neurofibroma	Benign	NF1	NEGATIVE	Normal	Preserved		1	1	1
**82**	16	m	L orbit	Plexiform neurofibroma	Benign	NF1	NEGATIVE	Normal	Preserved		1	1	1
**83**	39	M	R adrenal gland	Pheochromocytoma	Benign	NF1	NEGATIVE		Preserved		1	1	1
**84**	15	M	Spinal cord	Ganglioglioma	Low grade	NF1	NEGATIVE				1	1	1
85	54	M	brain	Anaplastic astrocytoma	High grade	NF1	NEGATIVE		Preserved			1	1
**85**	54	M	pelvis	GIST	High grade	NF1	NEGATIVE	Normal	Preserved		1	1	1
**86**	26	M	R conjunctiva	Plexiform neurofibroma	Benign	NF1	NEGATIVE	Normal			1	1	2
86	25	M	Orbit	Plexiform neurofibroma	Benign	NF1	NEGATIVE	Normal					1
**87**	64	M	Lumber nerve root	MPNST	High grade	sporadic	NEGATIVE	Short			1	1	1
**88**	57	F	L adrenal gland	Pheochromocytoma	Benign	NF1	NEGATIVE		Loss		1	1	1
**89**	38	M	L femoral nerve	Atypical neurofibroma	Benign	NF1	NEGATIVE	Normal	Preserved		1	1	1
**90**	64	F	L upper lid	Neurofibroma	Benign	NF1	NEGATIVE	Normal	Preserved		1	1	1
**91**	49	M	L peroneal nerve	Atypical neurofibroma	Benign	NF1	NEGATIVE	Normal	Preserved		1	1	1
**92**	59	F	Eyelid	Neurofibroma	Benign	NF1	NEGATIVE		Preserved		1	1	1
**93**	54	F	small bowel	GIST	High grade	NF1	NEGATIVE	Normal	Preserved		1	1	1
93	54	F	Ovaries (disseminated)	Ovarian serous carcinoma	High grade	NF1	NEGATIVE		Preserved			1	1
94	44	F	Pelvis	Atypical neurofibroma	Benign	NF1	NEGATIVE	Normal	Preserved			1	1
**94**	44	F	Sciatic nerve	MPNST	High grade	NF1	NEGATIVE	Normal	Preserved		1	1	1

**95**	15	F	Lumbar region	MPNST	High grade	NF1	NEGATIVE	Short	Preserved		1	1	1
**96**	18	F	supraclavicular	MPNST	High grade	NF1	NEGATIVE	Normal	Preserved		1	1	1
**97**	25	M	buttock	Plexiform neurofibroma	Benign	NF1	NEGATIVE	Normal	Preserved		1	1	1
98	39	F	Retroperitoneum	MPNST	High grade	NF1	NEGATIVE	Short		Preserved		1	1
**98**	37	F	retroperitoneum	Atypical neurofibroma	Benign	NF1	NEGATIVE	Normal	Preserved		1	1	1
98	40	F	brain	MPNST	High grade	NF1	NEGATIVE	Short	Preserved			1	3
98	39	F	retroperitoneum	MPNST	High grade	NF1	NEGATIVE	Short					1
**99**	60	M	Lung	MPNST	High grade	sporadic	NEGATIVE	Short	Preserved		1	1	3
99	58	M	inguinal region	MPNST	High grade	sporadic	NEGATIVE	Short				1	3
**100**	79	F	L eyelid	Neurofibroma	Benign	NF1	NEGATIVE	Normal	Preserved		1	1	1
**101**	42	F	R plexus	Plexiform neurofibroma	Benign	NF1	NEGATIVE	Normal	Preserved		1	1	1
**102**	50	F	skin	Neurofibroma	Benign	NF1	NEGATIVE	Normal	Preserved		1	1	1
102	62	F	L orbit	Neurofibroma	Benign	NF1	NEGATIVE	Normal	Preserved			1	1
**103**	10	M	Femoral nerve	MPNST	High grade	NF1	NEGATIVE	Long	Preserved		1	1	1
103	9	M	L thigh	Neurofibroma	Benign	NF1	Negative	Normal	Preserved			1	1
103	10	M	Femoral nerve	Plexiform neurofibroma	Benign	NF1			Preserved			1	1
**104**	27	M	L spinal	MPNST	High grade	Schwannomatosis	NEGATIVE	Normal	Preserved	Preserved	1	1	1
**105**	9	F	R lower lid	Plexiform neurofibroma	Benign	NF1	NEGATIVE	Normal	Preserved		1	1	1
**106**	17	F	L upper lid	Diffuse neurofibroma	Benign	NF1	NEGATIVE	Normal	Preserved		1	1	1
107	62	M	R brachial plexus	Atypical neurofibroma	Benign	NF1	NEGATIVE		Preserved			1	1
**107**	62	M	R brachial plexus	Neurofibroma	Benign	NF1	NEGATIVE		Preserved		1	1	1
**108**	32	M	skin	Neurofibroma	Benign	NF1	NEGATIVE	Normal			1	1	1
**109**	62	F	R index finger	Glomus tumor	Benign	NF1	NEGATIVE	Normal	Preserved		1	1	1
**110**	33	F	L posterior thigh/sciatic nerve	MPNST	High grade	NF1	POSITIVE	ALT	Loss	Equivocal	1	1	1
110	33	F	L posterior thigh/sciatic nerve	MPNST	High grade	NF1	POSITIVE	ALT	Loss	Preserved			1
110	33	F	L posterior thigh/sciatic nerve	Neurofibroma	Benign	NF1	NEGATIVE	Normal	Partial loss			1	1
**111**	2	F	L upper lid	Diffuse neurofibroma	Benign	NF1	NEGATIVE	Normal	Preserved		1		2
111	1	F	L orbit	Plexiform neurofibroma	Benign	NF1	NEGATIVE	Normal	Preserved			1	1
**112**	43	F	R orbit	Diffuse neurofibroma	Benign	NF1	NEGATIVE	Normal	Preserved		1	1	2
**113**	22	M	Pineal region	Pilocytic astrocytoma with anaplasia	High grade	NF1	POSITIVE	ALT	Loss		1	1	1
**114**	15	M	abdomen	Plexiform neurofibroma	Benign	NF1	NEGATIVE	Normal	Preserved		1	1	1
115	42	M	R chest wall	Neurofibroma	Benign	NF1	NEGATIVE	Normal	Preserved			1	1
115	42	M	L upper chest	Neurofibroma	Benign	NF1	NEGATIVE	Normal	Preserved			1	1
115	42	M	L center chest	Neurofibroma	Benign	NF1	NEGATIVE	Normal	Preserved			1	1
115	42	M	sternum	Neurofibroma	Benign	NF1	NEGATIVE	Normal	Preserved			1	1
115	42	M	L neck	Neurofibroma	Benign	NF1	NEGATIVE	Normal	Preserved			1	1

115	42	M	L lower abdomen	Neurofibroma	Benign	NF1	NEGATIVE	Normal	Preserved			1	1
115	42	M	L upper thigh	Neurofibroma	Benign	NF1	NEGATIVE	Normal	Preserved			1	1
115	42	M	buttocks	Neurofibroma	Benign	NF1	NEGATIVE	Normal	Preserved			1	1
115	42	M	L flank	Neurofibroma	Benign	NF1	NEGATIVE	Normal	Preserved			1	1
115	42	M	L flank	Neurofibroma	Benign	NF1	NEGATIVE	Normal	Preserved			1	1
115	42	M	Upper center back	Neurofibroma	Benign	NF1	NEGATIVE	Normal	Preserved			1	1
115	42	M	R upper back	Neurofibroma	Benign	NF1	NEGATIVE	Normal	Preserved			1	1
115	42	M	L posterior auricular	Neurofibroma+nevus	Benign	NF1	NEGATIVE	Normal	Preserved			1	1
115	42	M	L shoulder	Neurofibroma+nevus	Benign	NF1	NEGATIVE	Normal	Preserved			1	1
115	42	M	center upper chest	Neurofibroma+nevus	Benign	NF1	NEGATIVE	Normal	Preserved			1	1
115	42	M	center abdomen	Neurofibroma+nevus	Benign	NF1	NEGATIVE	Normal	Preserved			1	1
**115**	41	M	L adrenal	Pheochromocytoma	Benign	NF1	NEGATIVE				1	1	1
**116**	18	F	L thigh	Plexiform neurofibroma	Benign	NF1	NEGATIVE	Normal	Preserved		1	1	2
**117**	24	M	L paraspinal	MPNST	High grade	NF1	NEGATIVE	Normal	Preserved		1	1	1
117	22	M	R foot	Neurofibroma	Benign	NF1	NEGATIVE	Normal	Preserved			1	1
117	24	M	L paraspinal	Plexiform neurofibroma	Benign	NF1	NEGATIVE	Normal	Preserved				2
117	25	M	L paraspinal	Plexiform neurofibroma	Benign	NF1	NEGATIVE	Normal	Preserved				2
118	15	M	skin	Neurofibroma	Benign	NF1	NEGATIVE	Normal	Preserved			1	1
**118**	14	M	skin	Neurofibroma	Benign	NF1	NEGATIVE	Normal			1	1	1
**119**	39	M	small bowel	GIST	Low grade	NF1	NEGATIVE	Short			1	1	1
119	39	M	small bowel	GIST	Low grade	NF1	NEGATIVE	Short				1	1
119	39	M	small bowel	GIST	Low grade	NF1	NEGATIVE	Short				1	1
**120**	35	M	small bowel	GIST	Low grade	NF1	NEGATIVE	Normal	Preserved		1	1	1
**121**	46	F	skin	Neurofibroma	Benign	NF1	NEGATIVE	Normal	Preserved		1	1	1
**122**	44	M	Sciatic nerve	MPNST	High grade	NF1	NEGATIVE	Short	Preserved		1	1	2
122	41	M	Chest wall	Neurofibroma	Benign	NF1	NEGATIVE	Normal	Preserved			1	1
**123**	75	F	abdomen	GIST	High grade	NF1	NEGATIVE	Normal			1	1	1
**124**	35	F	L parieto-occipital lobe	Ganglioglioma	Low grade	NF1					1	1	1
**125**	8	F	R upper eyelid	Diffuse neurofibroma	Benign	NF1	NEGATIVE	Normal	Preserved		1	1	2
125	9	F	R upper eyelid	Diffuse neurofibroma	Benign	NF1	NEGATIVE	Normal					2
**126**	14	M	Brainstem	Diffuse Astrocytoma	Low grade	NF1	NEGATIVE	Normal	Preserved	Preserved	1	1	1
**127**	23	M	Sciatic nerve	MPNST	High grade	NF1	NEGATIVE	Normal			1	1	1
**128**	39	M	cerebellum	Diffuse Astrocytoma	Low grade	NF1	POSITIVE	ALT	Loss		1	1	1
**129**	20	F	Frontal lobe	SEGA-like astrocytoma	Low grade	NF1	NEGATIVE	Long	Preserved	Preserved	1	1	1
**130**	15	M	R frontal lobe	SEGA-like astrocytoma	Low grade	NF1	POSITIVE	ALT	Preserved	Preserved	1	1	1
**131**	17	M	L frontal lobe	SEGA-like astrocytoma	Low grade	NF1	NEGATIVE	Normal	Preserved	Preserved	1	1	1

**132**	24	F	brain	SEGA-like astrocytoma	Low grade	NF1	POSITIVE	ALT	Preserved	Preserved	1	1	1
**133**	53	F	Lateral ventricle	SEGA-like astrocytoma	Low grade	NF1	NEGATIVE	Long	Preserved		1	1	1
**134**	25	M	L frontal lobe	SEGA-like astrocytoma	High grade	NF1	POSITIVE	ALT	Aberrent	Preserved	1	1	1
**135**	9	M	Suprasellar	SEGA-like astrocytoma	Low grade	NF1	NEGATIVE		Preserved		1	1	1
**136**	12	F	cerebellum	Pilocytic astrocytoma with anaplasia	High grade	NF1	NEGATIVE	Long	Preserved		1	1	1
**137**	46	F	R temporal lobe	Pilocytic astrocytoma with anaplasia	High grade	NF1	NEGATIVE		Partial loss		1	1	1
**138**	29	M	cerebellum	Pilocytic astrocytoma with anaplasia	High grade	NF1	POSITIVE	ALT	Loss		1	1	1
**139**	46	M	L parietal lobe	Pilocytic astrocytoma with anaplasia	High grade	NF1	POSITIVE	ALT	Loss		1	1	1
**140**	4	F	Cerebellum	Glioblastoma with giant cell features	High grade	NF1	POSITIVE	ALT	Loss	Preserved	1	1	1
**141**	14	M	4th ventricle	Pilocytic astrocytoma	Low grade	NF1	NEGATIVE	Normal	Preserved	Preserved	1	1	1
**142**	48	F	Brain	Diffuse Astrocytoma	Low grade	NF1	POSITIVE	ALT	Partial loss	Preserved	1	1	1
**143**	34	M	Parietal lobe	Anaplastic pleomorphic xanthoastrocytoma	High grade	NF1	POSITIVE	ALT	Preserved	Preserved	1	1	1
**144**	19	F	Temporal lobe	Low grade astrocytoma	Low grade	NF1	NEGATIVE	Normal	Preserved	Preserved	1	1	1
**145**	5	M	Optic Nerve	Pilocytic astrocytoma	Low grade	NF1	NEGATIVE	Long	Preserved		1	1	1
**146**	20	F	L frontal lobe	Anaplastic pleomorphic xanthoastrocytoma	High grade	NF1	POSITIVE	ALT	Partial loss		1	1	1
**147**	4	F	Lateral ventricle	Pilocytic astrocytoma	High grade	NF1	NEGATIVE	Long	Preserved		1	1	1
147	2	F	Optic Nerve	Pilocytic astrocytoma	Low grade	NF1	NEGATIVE	Long	Preserved			1	1
147	4	F	Lateral ventricle	SEGA-like astrocytoma	Low grade	NF1	NEGATIVE						2
**148**	37	M	Brainstem	Pilocytic astrocytoma with atypical features	Low grade	NF1	POSITIVE	ALT	Loss		1	1	1
148	38	M	Brainstem	Pilocytic astrocytoma with atypical features	Low grade	NF1	POSITIVE	ALT	Loss				2
**149**	49	F	Frontal lobe	SEGA-like astrocytoma	High grade	NF1	NEGATIVE				1	1	1
**150**	25	F	Lateral ventricle	SEGA-like astrocytoma	Low grade	NF1	NEGATIVE	Normal	Preserved		1	1	1
**151**	46	F	Temporal lobe	SEGA-like astrocytoma	Low grade	NF1	NEGATIVE	Normal			1	1	1
**152**	17	M	L frontal lobe	Pilocytic astrocytoma	Low grade	NF1	NEGATIVE	Normal	Preserved		1	1	1
**153**	13	M	R frontal lobe	Pilocytic astrocytoma	Low grade	NF1	NEGATIVE	Long	Preserved		1	1	1
**154**	10	F	cerebellum	Pilocytic astrocytoma	Low grade	NF1	NEGATIVE	Normal	Preserved		1	1	1
**155**	2	M	L optic nerve	Pilocytic astrocytoma	Low grade	NF1	NEGATIVE	Normal	Partial loss		1	1	1
**156**	4	F	R thalamus	Pilocytic astrocytoma	Low grade	NF1	NEGATIVE		Preserved		1	1	2
**157**	21	F	L temporoparietal	Pilocytic astrocytoma	Low grade	NF1	NEGATIVE	Normal	Preserved		1	1	1
**158**	5	F	cerebellum	Pilocytic astrocytoma	Low grade	NF1	NEGATIVE	Long	Preserved		1	1	1
**159**	3	F	R optic nerve	Pilocytic astrocytoma	Low grade	NF1	NEGATIVE	Normal			1	1	1
**160**	5	M	L optic nerve	Pilocytic astrocytoma	Low grade	NF1	NEGATIVE	Normal	Preserved		1	1	1
**161**	5	F	L optic nerve	Pilocytic astrocytoma	Low grade	NF1	NEGATIVE	Long	Preserved		1	1	1
**162**	30	F	R lateral ventricle	Pilocytic astrocytoma	Low grade	NF1	NEGATIVE	Normal	Preserved		1	1	1
**163**	24	M	brainstem	Pilocytic astrocytoma	Low grade	NF1					1	1	1
164	33	F	Skin-chestwall	Cutaneous neurofibroma	Benign	NF1	NEGATIVE	Normal	Preserved			1	1

164	33	F	Skin-chestwall	Cutaneous neurofibroma	Benign	NF1	NEGATIVE	Normal	Preserved		1	1
164	33	F	Skin-chestwall	Cutaneous neurofibroma	Benign	NF1	NEGATIVE	Normal	Preserved		1	1
164	33	F	Skin-chestwall	Cutaneous neurofibroma	Benign	NF1	NEGATIVE	Normal	Preserved		1	1
164	33	F	Skin-chestwall	Cutaneous neurofibroma	Benign	NF1	NEGATIVE	Normal	Preserved		1	1
164	33	F	Skin-chestwall	Cutaneous neurofibroma	Benign	NF1	NEGATIVE	Normal	Preserved		1	1
164	33	F	Skin-chestwall	Cutaneous neurofibroma	Benign	NF1	NEGATIVE	Normal	Preserved		1	1
164	33	F	Skin-foot	Diffuse neurofibroma	Benign	NF1	NEGATIVE	Normal	Preserved		1	1
164	33	F	Skin-chestwall	Diffuse neurofibroma	Benign	NF1	NEGATIVE	Normal	Preserved		1	1
**164**	30	F	Buttock	Plexiform neurofibroma	Benign	NF1	NEGATIVE	Normal	Preserved	1	1	1
**165**	33	F	skin	Cutaneous neurofibroma	Benign	NF1	NEGATIVE	Normal	Preserved	1	1	1
165	33	F	Skin-cheek	Cutaneous neurofibroma	Benign	NF1	NEGATIVE	Normal	Preserved		1	1
165	33	F	Skin-hand	Cutaneous neurofibroma	Benign	NF1	NEGATIVE	Normal	Preserved		1	1
165	33	F	Skin-hand	Cutaneous neurofibroma	Benign	NF1	NEGATIVE	Normal	Preserved		1	1
165	33	F	Skin-back	Diffuse neurofibroma	Benign	NF1	NEGATIVE	Normal	Preserved		1	1
165	33	F	Skin-trunk	Diffuse neurofibroma	Benign	NF1	NEGATIVE	Normal	Preserved		1	1
**166**	46	F	Nerve root	Plexiform neurofibroma	Benign	NF1	NEGATIVE	Normal	Preserved	1	1	1
**167**	25	M	Midbrain	Pilocytic astrocytoma	Benign	NF1	NEGATIVE	Normal	Preserved	1	1	2
167	29	M	Posterior fossa	Pilocytic astrocytoma	Benign	NF1	NEGATIVE	Normal	Preserved			2
**168**	22	F	4th ventricle	Rosette forming glioneuronal tumor	Benign	NF1	NEGATIVE	Normal	Preserved	1	1	1
**169**	10	F	cerebellum	Pilocytic astrocytoma	Benign	NF1	NEGATIVE	Short	Preserved	1	1	1
**170**	20	M	L anterior neck	MPNST	High grade	NF1	NEGATIVE	Normal	Partial loss	1	1	1
**171**	31	M	Skin-R back	Cutaneous neurofibroma	Benign	NF1	NEGATIVE	Normal	Preserved	1	1	1
171	31	M	Skin-L arm	Cutaneous neurofibroma	Benign	NF1	NEGATIVE	Normal	Preserved		1	1
171	31	M	Skin-R shoulder	Cutaneous neurofibroma	Benign	NF1	NEGATIVE	Normal	Preserved		1	1
171	31	M	Skin-L lateral trunk	Cutaneous neurofibroma	Benign	NF1	NEGATIVE	Normal	Preserved		1	1
171	31	M	Skin-R chin	Cutaneous neurofibroma	Benign	NF1	NEGATIVE	Normal	Preserved		1	1
171	31	M	Soft tissue-L lateral trunk	Subcutaneous neurofibroma	Benign	NF1	NEGATIVE	Normal	Preserved		1	1
**172**	23	F	Skin-R shoulder	Cutaneous neurofibroma	Benign	NF1	NEGATIVE	Normal	Preserved	1	1	1
172	23	F	Skin-L shoulder	Cutaneous neurofibroma	Benign	NF1	NEGATIVE	Normal	Preserved		1	1
172	23	F	Skin-L middle back	Cutaneous neurofibroma	Benign	NF1	NEGATIVE	Normal	Preserved		1	1
172	23	F	Skin-Right middle back	Cutaneous neurofibroma	Benign	NF1	NEGATIVE	Normal	Preserved		1	1
172	23	F	Skin-inferior back	Cutaneous neurofibroma	Benign	NF1	NEGATIVE	Normal	Preserved		1	1
172	23	F	Skin-Middle back	Cutaneous neurofibroma	Benign	NF1	NEGATIVE	Normal	Preserved		1	1
**173**	30	M	Lateral ventricle	Anaplastic astrocytoma	High grade	NF1	POSITIVE	ALT	Loss	1	1	1
**174**	7	M	Skin-L eyelid	Juvenile xanthogranuloma	Benign	NF1	NEGATIVE	Short	Preserved	1	1	1
**175**	31	F	L forearm	Plexiform neurofibroma	Benign	NF1	NEGATIVE	Normal	Preserved	1	1	1

**176**	21	M	Paraspinal	Neurofibroma	Benign	NF1	NEGATIVE	Normal	Preserved	1	1	1
176	21	M	R thigh	Neurofibroma	Benign	NF1	NEGATIVE	Normal	Preserved		1	1
**177**	13	M	L orbit	Plexiform neurofibroma	Benign	NF1			Preserved	1	1	1
**178**	13	M	L eyelid	Plexiform neurofibroma	Benign	NF1	NEGATIVE	Normal	Preserved	1	1	1
**179**	9	F	L upper eyelid	Plexiform neurofibroma	Benign	NF1	NEGATIVE	Normal		1	1	1
**180**	14	M	L upper eyelid	Plexiform neurofibroma	Benign	NF1	NEGATIVE	Normal	Preserved	1	1	1
**181**	39	F	l upper eyelid	Cutaneous neurofibroma	Benign	NF1	NEGATIVE	Normal	Preserved	1	1	1
**182**	16	M	R orbit	Plexiform neurofibroma	Benign	NF1	NEGATIVE	Normal	Preserved	1	1	1
**183**	61	M	L upper eyelid	Localized neurofibroma	Benign	NF1	NEGATIVE	Normal	Preserved	1	1	1
**184**	23	F	L orbit	Diffuse neurofibroma	Benign	NF1	NEGATIVE	Normal	Preserved	1	1	1
**185**	43	F	Brain	Anaplastic astrocytoma	High grade	NF1	POSITIVE	ALT	Loss	1	1	1
**186**	37	F	skin-elbow	Cutaneous neurofibroma	Benign	NF1	NEGATIVE	Normal	Preserved	1	1	1
186			skin-elbow	Cutaneous neurofibroma	Benign	NF1	NEGATIVE	Normal	Preserved		1	1
186			Skin-chin	Cutaneous neurofibroma	Benign	NF1	NEGATIVE	Normal	Preserved		1	1
186			Skin-knee	Cutaneous neurofibroma	Benign	NF1	NEGATIVE	Normal	Preserved		1	1
186			Skin-knee	Cutaneous neurofibroma	Benign	NF1	NEGATIVE	Normal	Preserved		1	1
186			Skin-leg	Cutaneous neurofibroma	Benign	NF1	NEGATIVE	Normal	Preserved		1	1
**187**	1	M	R arm	Juvenile xanthogranuloma	Benign	NF1	NEGATIVE	Normal	Preserved	1	1	1
187				Juvenile xanthogranuloma	Benign	NF1	NEGATIVE	Normal	Preserved		1	1
**188**	34	M	Sella	Glioblastoma	High grade	NF1	POSITIVE	ALT	Preserved	1	1	1
189	44	F	retroperitoneum	MPNST	High grade	NF1	NEGATIVE	Short	Preserved		1	1
189	44	F	L hip-skin	Diffuse neurofibroma	Benign	NF1	NEGATIVE	Normal			1	1
189	44	F	R leg-skin	Diffuse neurofibroma	Benign	NF1	NEGATIVE	Normal			1	1
**189**	44	F	Liver	Plexiform neurofibroma	Benign	NF1	NEGATIVE	Normal		1	1	1
189	44	F	L adrenal-	Plexiform neurofibroma	Benign	NF1	NEGATIVE	Normal			1	1
**190**	40	M	R frontal lobe	Diffuse Astrocytoma	Low grade	NF1			Partial loss	1	1	1
**191**	18	M	Pelvis	MPNST	High grade	NF1	NEGATIVE	Short	Preserved	1	1	1
191	18	M	Pelvis	Plexiform neurofibroma	Benign	NF1	NEGATIVE	Normal	Preserved		1	1
**192**	58	M	R arm	Cutaneous neurofibroma	Benign	NF1	NEGATIVE	Normal	Preserved	1	1	1
192	58	M	R forehead	Cutaneous neurofibroma	Benign	NF1	NEGATIVE	Normal	Preserved		1	1
193	53	M	L thigh	ANNUBP	Benign	NF1	NEGATIVE	Normal	Preserved		1	1
193	53	M	Tibial nerve	Schwannoma	Benign	NF1	NEGATIVE	Normal	Preserved		1	1
**193**	55	M	Chest wall	MPNST	High grade	NF1	NEGATIVE	Short		1	1	1
**194**	53	F	Lung	MPNST	High grade	NF1	NEGATIVE	Short	Preserved	1	1	3
**195**	25	F	R neck	Plexiform neurofibroma	Benign	NF1	NEGATIVE	Normal	Preserved	1	1	1
196	41	M	L arm	Plexiform neurofibroma	Benign	NF1	NEGATIVE	Normal	Partial loss		1	1

196	41	M	R forearma	Cutaneous neurofibroma	Benign	NF1	NEGATIVE	Normal	Preserved			1	1
**196**	41	M	R median nerve	Localized intraneural neurofibroma	Benign	NF1	NEGATIVE	Normal	Preserved		1	1	1
196	41	M		Plexiform neurofibroma	Benign	NF1	NEGATIVE	Normal				1	1
**197**	59	M	duodenum	Neuroendocrine tumor		NF1	NEGATIVE	Normal			1	1	1
**198**	52	F	ampulla	Neuroendocrine tumor	Low grade	NF1	NEGATIVE	Short			1	1	1
**199**	61	F	duodenum	Neuroendocrine tumor	Low grade	NF1	NEGATIVE	Normal			1	1	1
**200**	46	M	duodenum	Neuroendocrine tumor		NF1	NEGATIVE	Short			1	1	1
**201**	40	M	ampulla	Neuroendocrine tumor	Low grade	NF1	NEGATIVE	Short			1	1	1
**202**	55	M	ampulla	Neuroendocrine tumor	Low grade	NF1	NEGATIVE	Short			1	1	1
**203**	42	F	retroperitoneal	MPNST	High grade	NF1			Preserved	Preserved	1	1	
**204**	29	F	thigh	MPNST	High grade	NF1	POSITIVE	ALT	Preserved	Preserved	1	1	
**205**	40	M	retroperitoneal	MPNST	Low grade	NF1	NEGATIVE		Preserved	Preserved	1	1	
**206**	44	M	sacrum	MPNST	High grade	NF1	NEGATIVE		Preserved	Preserved	1	1	
**207**	31	M	thigh	MPNST	High grade	NF1	NEGATIVE			Preserved	1	1	
**208**	45	F	thigh	MPNST	High grade	NF1	POSITIVE	ALT	Preserved	Preserved	1	1	
**209**	49	F	spine	MPNST	High grade	NF1	NEGATIVE		Preserved	Preserved	1	1	
**210**	43	F	brachial plexus	MPNST	High grade	NF1	NEGATIVE		Preserved	Preserved	1	1	
**211**	11	M	retroperitoneal	MPNST	High grade	NF1	NEGATIVE		Partial loss	Preserved	1	1	
**212**	43	M	retroperitoneal	MPNST	High grade	NF1	POSITIVE	ALT	Partial loss	Preserved	1	1	
**213**	27	F	retroperitoneal	MPNST	High grade	NF1	NEGATIVE		Partial loss	Preserved	1	1	
**214**	60	F	face	MPNST	High grade	NF1	NEGATIVE		Preserved	Preserved	1	1	
**215**	23	M	arm	MPNST	High grade	NF1	NEGATIVE		Preserved	Preserved	1	1	
**216**	31	M	thigh	MPNST	High grade	NF1	NEGATIVE		Partial loss	Preserved	1	1	
**217**	47	F	spine	MPNST	High grade	NF1	NEGATIVE		Preserved	Preserved	1	1	
**218**	34	F	tibia	MPNST	Low grade	NF1	NEGATIVE		Preserved	Preserved	1	1	
**219**	22	M	spine	MPNST	Low grade	NF1	NEGATIVE		Preserved	Preserved	1	1	
**220**	49	F	sacrum	MPNST	High grade	NF1	POSITIVE	ALT	Preserved	Preserved	1	1	
**221**	33	F	back	MPNST	High grade	NF1	NEGATIVE		Preserved	Preserved	1	1	
**222**	43	M	forearm	MPNST	High grade	NF1	NEGATIVE		Partial loss	Preserved	1	1	
**223**	35	F	neck	MPNST	High grade	NF1	NEGATIVE		Partial loss	Preserved	1	1	
**224**	33	M	shoulder	MPNST	High grade	NF1	NEGATIVE		Preserved	Preserved	1	1	
**225**	38	F	sacrum	MPNST	High grade	NF1	NEGATIVE		Partial loss	Preserved	1	1	
**226**	29	M	neck	MPNST	High grade	NF1	NEGATIVE		Preserved	Preserved	1	1	
**227**	37	M	spine	MPNST	High grade	NF1	NEGATIVE			Preserved	1	1	
**228**	13	F	mediastinum	MPNST	High grade	NF1	NEGATIVE		Preserved	Preserved	1	1	
**229**	42	F	sacrum	MPNST	High grade	Sporadic	NEGATIVE		Preserved	Preserved	1	1	

**230**	33	M	mediastinum	MPNST	High grade	Sporadic	POSITIVE	ALT	Loss	Preserved	1	1	
**231**	35	M	thigh	MPNST	High grade	.	NEGATIVE		Preserved	Preserved	1	1	
**232**	53	F	brachial plexus	MPNST	High grade	Sporadic	NEGATIVE		Partial loss	Preserved	1	1	
**233**	65	F	chest wall	MPNST	High grade	Sporadic					1	1	
**234**	49	M	brachial plexus	MPNST	High grade	Sporadic	NEGATIVE		Partial loss	Partial loss	1	1	
**235**	43	F	thigh	MPNST	High grade	Sporadic					1	1	
**236**	57	F	thigh	MPNST	High grade	Sporadic	NEGATIVE		Preserved	Preserved	1	1	
**237**	72	F	thigh	MPNST	High grade	Sporadic	NEGATIVE		Preserved	Partial loss	1	1	
**238**	23	F	calf	MPNST	High grade	Sporadic	NEGATIVE		Preserved	Preserved	1	1	
**239**	36	F	retroperitoneal	MPNST	High grade	Sporadic	POSITIVE	ALT	Preserved	Preserved	1	1	
**240**	32	F	retroperitoneal	MPNST	High grade	Sporadic	NEGATIVE		Preserved	Preserved	1	1	
**241**	49	M	ST-neck	MPNST	High grade		NEGATIVE				1	1	1
**242**	28	F	ST-neck	MPNST	High grade		NEGATIVE				1	1	1
**243**	27	F	ST-chest wall	MPNST	High grade		POSITIVE	ALT			1	1	1
**244**	77	M	ST-axilla	MPNST	Low grade		NEGATIVE				1	1	1
**245**	37	F	ST-pelvis	MPNST	High grade		NEGATIVE				1	1	1
**246**	44	M	ST-neck	MPNST	High grade		NEGATIVE				1	1	1
**247**	66	M	Lung-met	MPNST	High grade		NEGATIVE				1	1	3
**248**	43	F	ST-brachial p.	MPNST	High grade		NEGATIVE				1	1	1
**249**	37	F	ST-pelvis	MPNST	High grade		NEGATIVE				1	1	2
**250**	39	M	ST-pelvis	MPNST	High grade		NEGATIVE				1	1	1
**251**	76	M	ST-paraspinal	MPNST	High grade		POSITIVE	ALT			1	1	1
**252**	73	M	ST-shoulder	MPNST	High grade		NEGATIVE				1	1	1
**253**	34	F	ST-thigh	MPNST	High grade		NEGATIVE				1	1	1
**254**	51	F	ST-chest wall	MPNST	High grade		NEGATIVE				1	1	1
**255**	36	F	ST-chest wall	MPNST	High grade		NEGATIVE				1	1	1
**256**	59	M	ST-arm	MPNST	High grade		POSITIVE	ALT			1	1	1
**257**	38	M	ST-pelvis	MPNST	High grade		NEGATIVE				1	1	1
**258**	26	F	ST-arm	MPNST	High grade		NEGATIVE				1	1	2
**259**	56	M	ST-chest wall	MPNST	High grade		POSITIVE	ALT			1	1	1
**260**	74	F	ST-thigh	MPNST	High grade		POSITIVE	ALT			1	1	2
**261**	23	M	ST-mediastinum	MPNST	High grade		NEGATIVE				1	1	1
**262**	23	F	ST-infratemp.	MPNST	High grade		NEGATIVE				1	1	1
**263**	16	F	Lymph node	MPNST	High grade		NEGATIVE				1	1	3
**264**	51	F	Lymph node	MPNST	High grade		POSITIVE	ALT			1	1	3
**265**	15	M	ST-inguinal	MPNST	High grade	NF1	NEGATIVE				1	1	1

**266**	43	M	ST-paraspinal	MPNST	High grade		POSITIVE	ALT	1	1	1
**267**	32	M	ST-chest wall	MPNST	High grade	NF1	NEGATIVE		1	1	1
**268**	24	F	ST-pelvis	MPNST	High grade	NF1	NEGATIVE		1	1	1
**269**	77	F	ST-orbit	MPNST	Low grade		POSITIVE	ALT	1	1	1
**270**	29	F	ST-thigh	MPNST	High grade	NF1	NEGATIVE		1	1	1
**271**	34	F	ST-leg	MPNST	High grade		NEGATIVE		1	1	1
**272**	31	M	ST-retroperitoneum	MPNST	High grade	NF1	NEGATIVE		1	1	1
**273**	33	M	Skin-clavicle	MPNST	Low grade		NEGATIVE		1	1	1
**274**	71	F	ST-abd.	MPNST	High grade	NF1	NEGATIVE		1	1	1
**275**	21	F	ST-paraspinal	MPNST	High grade	NF1	NEGATIVE		1	1	1
**276**	16	M	ST-mediast.	MPNST	Low grade	NF1	NEGATIVE		1	1	1
**277**	33	F	ST-brach. pl.	MPNST	High grade	NF1	NEGATIVE		1	1	1
**278**	48	F	ST-buttock	MPNST	High grade		NEGATIVE		1	1	1
**279**	65	F	ST-peroneal n.	MPNST	Low grade		NEGATIVE		1	1	1
**280**	3	M	ST-forearm	MPNST	High grade		NEGATIVE		1	1	1
**281**	48	M	Diaphragm	MPNST	High grade		NEGATIVE		1	1	1
**282**	52	M	ST-paraspinal	MPNST	High grade		NEGATIVE		1	1	1
**283**	62	F	ST-brach. pl.	MPNST	High grade		NEGATIVE		1	1	1
**284**	24	M	ST-neck	MPNST	High grade	NF1	NEGATIVE		1	1	1
**285**	68	M	ST-arm	MPNST	High grade		NEGATIVE		1	1	1
**286**	42	F	ST-brach. pl.	MPNST	High grade		NEGATIVE		1	1	1
**287**	47	M	ST-mediast.	MPNST	High grade		NEGATIVE		1	1	1
**288**	25	M	ST-retroperit.	MPNST	High grade	NF1	NEGATIVE		1	1	1
**289**	43	M	ST-back	MPNST	High grade	NF1	NEGATIVE		1	1	1
**290**	33	F	ST-back	MPNST	High grade	NF1	NEGATIVE		1	1	1
**291**	40	M	ST-back	MPNST	High grade		NEGATIVE		1	1	1
**292**	31	F	ST-thigh	MPNST	High grade	NF1	NEGATIVE		1	1	1
**293**	33	M	ST-retroperit.	MPNST	High grade	NF1	POSITIVE	ALT	1	1	1
293	33	M	ST-retroperit.	MPNST	High grade	NF1	POSITIVE	ALT			2
**294**	47	F	ST-brach. pl.	Epithelioid MPNST	High grade		NEGATIVE		1	1	1
**295**	74	F	ST-brach. pl.	MPNST	High grade		NEGATIVE		1	1	1
**296**	49	M	ST-abdomen	MPNST	High grade	NF1	NEGATIVE		1	1	1
296	51	M	ST-axilla	MPNST	High grade	NF1	NEGATIVE			1	1
296	51	M	ST-axilla	MPNST	High grade	NF1	NEGATIVE				2
**297**	30	M	ST-neck	MPNST	High grade		NEGATIVE		1	1	1
297	30	M	ST-neck	MPNST	High grade		NEGATIVE				2

**298**	38	F	ST-femoral	MPNST	High grade		NEGATIVE		1	1	1
298	43	F	ST-femoral	MPNST	High grade		NEGATIVE				
**299**	46	M	ST-pelvis	MPNST	Low grade		NEGATIVE		1	1	1
300	41	F	ST-thigh	MPNST	High grade		NEGATIVE				2
**300**	37	F	ST-thigh	MPNST	Low grade		NEGATIVE		1	1	1
300	39	F	ST-groin	MPNST	Low grade		NEGATIVE				2
301	25	M	ST-chest wall	MPNST	High grade	NF1	NEGATIVE			1	1
**301**	21	M	ST-sciatic n.	MPNST	Low grade	NF1			1	1	1
**302**	46	F	ST-thigh	MPNST	High grade		NEGATIVE		1	1	1
302	46	F	Lung-met	MPNST	High grade		NEGATIVE			1	3
**303**	58	M	ST-femoral	MPNST	High grade		POSITIVE	ALT	1	1	1
303	58	M	ST-femoral	MPNST	High grade		POSITIVE	ALT			2
**304**	81	F	ST-leg	MPNST	High grade		POSITIVE	ALT	1	1	1
304	86	F	ST-leg	MPNST	High grade		POSITIVE	ALT			2
304	86	F	ST-groin	MPNST	High grade		POSITIVE	ALT			3
**305**	42	M	ST-sciatic n.	MPNST	High grade		NEGATIVE		1	1	1
**306**	60	F	ST-infratemp.	MPNST	High grade		NEGATIVE		1	1	1
306	60	F	ST-shoulder-met	MPNST	High grade		NEGATIVE				3
**307**	49	F	ST-arm	MPNST	High grade		POSITIVE	ALT	1	1	1
307	49	F	Pleura-met	MPNST	High grade		NEGATIVE			1	3
308	18	M	ST-buttock	MPNST	High grade		NEGATIVE				2
**308**	17	M	ST-buttock	MPNST	Low grade		NEGATIVE		1	1	2
**309**	35	M	ST-chest wall	MPNST	High grade		NEGATIVE		1	1	1
309	37	M	Bone-met	MPNST	High grade		NEGATIVE			1	2
**310**	46	F	ST-arm	MPNST	High grade		POSITIVE	ALT	1	1	1
**311**	23	F	ST-thigh	MPNST	High grade	NF1	NEGATIVE		1	1	1
**312**	48	M	ST-buttock	MPNST	High grade		POSITIVE	ALT	1	1	1
313	59	F	ST-chest wall	MPNST	High grade	NF1	NEGATIVE			1	1
**314**	68	M	ST-thigh	MPNST	High grade		POSITIVE	ALT	1	1	1
**315**	21	F	ST-skull base	MPNST	Low grade	NF1	NEGATIVE		1	1	1
316	34	M	ST-arm	MPNST	High grade		NEGATIVE				1
316	34	M	ST-paraspinal	MPNST	High grade		NEGATIVE				1
**317**	1	F	ST-paraspinal	MPNST	High grade		NEGATIVE		1	1	1
318	9	M	ST-leg	MPNST	High grade		NEGATIVE				1
319	17	F	Lung	MPNST	High grade	NF1					3
**319**	16	F	ST-thigh	MPNST	High grade	NF1	NEGATIVE		1	1	1

320	13	F	Lung + parasp.	MPNST	High grade	NF1	NEGATIVE				1	3
**321**	12	F	ST-back	MPNST	High grade	NF1	NEGATIVE			1	1	1
322	81	M	ST-thigh	Epithelioid MPNST	High grade		POSITIVE	ALT				1
**323**	24	F	ST-abd.	MPNST	High grade	NF1	POSITIVE	ALT		1	1	1
323	24	F	ST-abd.	MPNST	High grade	NF1	POSITIVE	ALT				2
323	25	F	Bowel	MPNST	High grade	NF1	POSITIVE	ALT				2
323	26	F	Bowel	MPNST	High grade	NF1	POSITIVE	ALT				2
**324**	11	F	ST-thigh	MPNST	Low grade	NF1	NEGATIVE			1	1	1
324	13	F	ST-thigh	MPNST	Low grade	NF1	NEGATIVE					2
325	36	F	ST-brach. pl.	MPNST	High grade		NEGATIVE					1
**326**	17	F	ST-paraspinal	MPNST	High grade		NEGATIVE			1	1	1
326	17	F	ST-paraspinal	MPNST	High grade		NEGATIVE					2
326	18	F	ST-paraspinal	MPNST	High grade		NEGATIVE					2
326	18	F	ST-paraspinal	MPNST	High grade		NEGATIVE					2
326	18	F	ST-paraspinal	MPNST	High grade		NEGATIVE					2
**327**	37	M	buttock	Massive soft tissue neurofibroma	Benign	NF1	NEGATIVE	Normal	Preserved	1	1	1
**328**	10	F	Scalp	MPNST with divergent differentiation	High grade	NF1	NEGATIVE	Normal	Preserved	1	1	1
**329**	14	M	third ventricle	Pilocytic astrocytoma	Low grade	NF1	NEGATIVE	Long	Preserved	1	1	1
**330**	24	M	cerebellum	Pilocytic astrocytoma with anaplasia	High grade	NF1	POSITIVE	ALT	Loss	1	1	1
**331**	11	F	R occipital lobe	Pilocytic astrocytoma with anaplasia	High grade	NF1	NEGATIVE		Preserved	1	1	1
**332**	18	M	L parietal lobe	Pilocytic astrocytoma with anaplasia	High grade	NF1	NEGATIVE		Preserved	1	1	1
**333**	21	M	small bowel	GIST	Low grade	NF1	NEGATIVE	Normal		1	1	1
**334**	35	M	CP angle	Pilocytic astrocytoma with anaplasia	High grade	NF1	POSITIVE	ALT	Loss	1	1	1
335	18	F	breast	Malignant Phylloides tumor	High grade	NF1	POSITIVE	ALT	Preserved			
**336**	79	F	Stomach	Gastric Schwannoma	Benign	NF1	NEGATIVE	Normal	Preserved	1	1	1
337	79	F	Stomach	GIST	Low grade	NF1	NEGATIVE	Short	Preserved		1	1

Table S2. Next generation sequencing results of ALT positive NF1-associated gliomas and MPNST (n=9)

ID	Chr:Pos	Base Change	Region	Gene	CDS	AA_change	Alt+,Alt- /Ref+,Ref-	%VAF	%RAF	Avg Cov
CASE 78-MPNST	chr17:29508438	A>G	splicing	NF1	NM_000267 : c.587-2A>G		27,11/31,23	41.3	58.7	512.31
CASE 78-MPNST	chr4:87968606	C>G	exonic	AFF1	NM_001166693.2 : c.C919G	p.Q307E	9,15/111,121	9.38	90.62	1975.36
CASE 78-MPNST	chr18:50450208	G>A	exonic	DCC	NM_005215 : c.G829A	p.E277K	9,16/22,20	37.31	62.69	553.58
CASE 78-MPNST	chr11:108788634	G>GTGATGA	exonic	DDX10	NM_004398 : c.2363_2364insTGATGA	p.D788_G789insDD	18,11/57,42	22.66	77.34	544.29
CASE 78-MPNST	chr4:153253751	C>T	exonic	FBXW7	NM_033632 : c.G982A	p.E328K	7,7/52,61	11.02	88.98	682.26
CASE 78-MPNST	chr5:180053190	G>C	exonic	FLT4	NM_182925 : c.C1179G	p.S393R	46,49/42,49	51.08	48.92	888.36
CASE 78-MPNST	chr2:240002870	C>T	exonic	HDAC4	NM_006037 : c.G2656A	p.V886M	69,60/49,51	56.33	43.67	988.56
CASE 78-MPNST	chr17:45377872	C>T	exonic	ITGB3	NM_000212 : c.C1942T	p.R648W	32,32/34,28	50.79	49.21	1071.05
CASE 78-MPNST	chr6:15496478	C>T	exonic	JARID2	NM_004973 : c.C1022T	p.T341M	32,31/36,40	45.32	54.68	1499.22
CASE 78-MPNST	chr19:36214639	C>T	exonic	KMT2B	NM_014727 : c.C3065T	p.T1022M	58,31/38,32	55.97	44.03	633.4
CASE 78-MPNST	chr3:46480815	C>T	exonic	LTF	NM_002343 : c.G1880A	p.R627H	51,33/42,30	53.85	46.15	844.98
CASE 78-MPNST	chr17:5442764	C>G	exonic	NLRP1	NM_033004 : c.G2841C	p.R947S	67,56/45,39	59.42	40.58	834.55
CASE 78-MPNST	chr1:186644489	C>T	exonic	PTGS2	NM_000963 : c.G1297A	p.V433I	22,28/23,37	45.45	54.55	868.82
CASE 78-MPNST	chr8:145741989	G>A	exonic	RECQL4	NM_004260 : c.C514T	p.L172F	46,58/45,42	54.45	45.55	1068.43
CASE 78-MPNST	chr8:145743144	C>T	exonic	RECQL4	NM_004260 : c.G25A	p.E9K	4,11/3,10	53.57	46.43	107.88
CASE 78-MPNST	chr18:42281466	G>A	exonic	SETBP1	NM_015559 : c.G155A	p.G52E	72,66/54,57	55.42	44.58	1090.07
CASE 78-MPNST	chr1:144994694	T>C	exonic	PDE4DIP	NM_014644 : c.38A>G	p.N13S	41,42/224,231	15.43	84.57	2336.7

ID	Chr:Pos	Base Change	Region	Gene	CDS	AA_change	Alt+,Alt- /Ref+,Ref-	%VAF	%RAF	Avg Cov
CASE 53-MPNST	chrX:76937963	G>C	exonic	ATRX	NM_000489.4: c.C2785G	p.Q929E	244,313/129,134	67.93	32.07	1223.62
CASE 53-MPNST	chr2:29606597	C>A	splicing	ALK	NM_004304.4 : c.1282+1G>T	p.?	194,120/316,235	36.3	63.7	958.39
CASE 53-MPNST	chr17:29585446	T>TCACC	exonic	NF1	NM_000267.3 : c.4200_4204insCACC	p.Y1401Tfs*2	198,235/426,547	30.8	69.2	1067.92
CASE 53-MPNST	chr17:7577139	G>A	exonic	TP53	NM_000546.5 : c.C799T	p.R267W	77,184/115,225	43.43	56.57	1135.22
CASE 53-MPNST	chr16:2134269	C>T	exonic	TSC2	NM_000548.4 : c.C4046T	p.A1349V	15,10/201,172	6.28	93.72	558.6
CASE 53-MPNST	chrX:39933843	G>T	exonic	BCOR	NM_017745.5 : c.C756A	p.Y252*	135,153/294,260	34.2	65.8	1123.94
CASE 53-MPNST	chr12:121881950	C>G	exonic	KDM2B	NM_032590.4 : c.G2316C	p.E772D	286,246/238,226	53.41	46.59	920.01
CASE 53-MPNST	chr5:176637570	G>A	exonic	NSD1	NM_022455.4 : c.G2170A	p.E724K	132,110/390,415	23.11	76.89	1342.3
CASE 53-MPNST	chr8:48815306	C>T	exonic	PRKDC	NM_006904.6 : c.G3092A	p.R1031Q	59,82/273,350	18.46	81.54	964.06
CASE 53-MPNST	chr9			CDKN2A/B		Homozgyous loss				
CASE 53-MPNST	chr17			TP53		Heterozygous loss				

ID	Chr:Pos	Base Change	Region	Gene	CDS	AA_change	Alt+,Alt- /Ref+,Ref-	%VAF	%RAF	Avg Cov
CASE 110-MPNST	chr1:40366764	GAGCGAGCCGGTCGCTCACAGCTCTCTCC>G	exonic	MYCL	NM_001033082.2 : c.405_432del	p.E136Lfs*59	5,5/70,69	6.71	93.29	555.99

CASE 110-MPNST	chr11:108546344	G>A	exonic	DDX10	NM_004398 : c.G269A	p.R90H	12,5/17,13	36.17	63.83	246.24
CASE 110-MPNST	chr11:119167694	G>T	exonic	CBL	NM_005188 : c.G2103T	p.M701I	16,11/11,19	47.37	52.63	371.64
CASE 110-MPNST	chr11:94180441	C>T	exonic	MRE11A	NM_005591 : c.G1727A	p.R576Q	15,14/20,15	45.31	54.69	536.91
CASE 110-MPNST	chr13:103492133	C>T	exonic	BIVM-ERCC5	NM_001204425 : c.C1430T	p.S477L	17,22/18,23	48.75	51.25	998.43
CASE 110-MPNST	chr15:91346819	G>A	exonic	BLM	NM_000057 : c.G3427A	p.E1143K	13,14/23,25	36	64	571.56
CASE 110-MPNST	chr16:2983556	G>A	exonic	FLYWCH1	NM_032296 : c.G1219A	p.E407K	20,21/42,48	31.3	68.7	925.6
CASE 110-MPNST	chr16:68772311	A>G	exonic	CDH1	NM_004360 : c.A160G	p.R54G	25,20/21,34	45	55	386.4
CASE 110-MPNST	chr16:9857094	T>C	exonic	GRIN2A	NM_000833 : c.A4307G	p.N1436S	9,8/17,19	32.08	67.92	620.08
CASE 110-MPNST	chr17:29677200	G>A	splicing	NF1	NM_000267 : c.7259-1G>A		12,10/8,8	57.89	42.11	522.21
CASE 110-MPNST	chr17:5463285	G>A	exonic	NLRP1	NM_033004 : c.C731T	p.A244V	7,7/42,44	14	86	687.81
CASE 110-MPNST	chr17:75478271	C>T	exonic	SEPT9	NM_001113491.1 : c.767C>T	p.P256L	85,78/88,82	48.95	51.05	1507.52
CASE 110-MPNST	chr17:76212770	G>A	exonic	BIRC5	NM_001168 : c.G247A	p.G83S	21,27/27,48	39.02	60.98	756.65
CASE 110-MPNST	chr18:59221719	G>T	exonic	CDH20	NM_031891 : c.G2197T	p.A733S	31,29/49,46	38.71	61.29	940.94
CASE 110-MPNST	chr2:202123045	G>T	exonic	CASP8	NM_001080125 : c.G91T	p.V31L	26,24/64,52	30.12	69.88	848.56
CASE 110-MPNST	chr2:216288177	T>C	exonic	FN1	NM_212482 : c.A1289G	p.N430S	6,7/20,21	24.07	75.93	378.7
CASE 110-MPNST	chr2:216299526	T>C	exonic	FN1	NM_212482 : c.A170G	p.K57R	20,42/13,13	70.45	29.55	806.6
CASE 110-MPNST	chr3:30691871	GA>G	exonic	TGFBR2	NM_003242 : c.374delA	p.K128Sfs*35	10,15/54,62	17.73	82.27	1590.71
CASE 110-MPNST	chr3:52643618	G>A	exonic	PBRM1	NM_018313 : c.C2278T	p.R760C	7,7/18,21	26.42	73.58	800.04
CASE 110-MPNST	chr5:149503816	C>T	exonic	PDGFRB	NM_002609 : c.G2020A	p.G674R	26,28/40,44	39.13	60.87	853.42
CASE 110-MPNST	chr5:180036005	G>C	exonic	FLT4	NM_182925 : c.C3856G	p.Q1286E	8,8/29,29	21.62	78.38	445.09
CASE 110-MPNST	chr5:56176912	T>C	exonic	MAP3K1	NM_005921 : c.T2182C	p.S728P	17,16/24,18	44	56	682.1
CASE 110-MPNST	chr6:106553314	G>A	exonic	PRDM1	NM_001198 : c.G1279A	p.A427T	29,26/50,51	35.26	64.74	1750.92
CASE 110-MPNST	chr6:51771137	C>G	exonic	PKHD1	NM_138694.3 : c.G6684C	p.E2228D	12,14/14,12	50	50	271.32
CASE 110-MPNST	chr7:80299305	AGGTATTGCAGTTCTTTTCTTCT>A	exonic	CD36	NM_000072 : c.787_808del	p.V263Ifs*16	9,10/26,32	24.68	75.32	712.74
CASE 110-MPNST	chr8:113657421	C>T	exonic	CSMD3	NM_198123 : c.3227G>A	p.G1076E	14,22/16,29	44.44	55.56	426.28
CASE 110-MPNST	chr8:30916743	C>A	exonic	WRN	NM_000553 : c.C171A	p.Y57X	13,17/21,23	40.54	59.46	909.72
CASE 110-MPNST	chr9:21971208	C>G	splicing	CDKN2A	NM_000077 : c.151-1G>C		9,11/14,27	32.79	67.21	424.69
CASE 110-MPNST	chrX:76939216	TC>T	exonic	ATRX	NM_000489 : c.1531delG	p.E511Kfs*3	8,8/22,19	28.07	71.93	750.5

**ID**	**Chr:Pos**	**Base Change**	**Region**	**Gene**	**CDS**	**AA_change**	**Alt+,Alt-/Ref+,Ref-**	**%VAF**	**%RAF**	**Avg Cov**
Case 130-GLIOMA	chr2:100210342	CCTCAGCC>GC	exonic	AFF3	NM_1025108: c.1856_1863delinsGC	p.T619_A621delinsS	6,6/41,33	14	86	514.98
Case 130-GLIOMA	chr19:6586291	T>C	exonic	CD70	NM_001252 : c.A322G	p.I108V	22,38/67,56	32.79	67.21	287.15
Case 130-GLIOMA	chr16:68842734	C>T	exonic	CDH1	NM_004360 : c.C670T	p.R224C	53,57/21,29	68.75	31.25	516.11
Case 130-GLIOMA	chr7:142562051	CCCT>C	exonic	EPHB6	NM_004445 : c.497_499delCCT	p.S177del	28,28/78,76	26.67	73.33	542.61
Case 130-GLIOMA	chrX:48652297	G>A	exonic	GATA1	NM_002049 : c.G968A	p.G323D	8,6/73,79	8.43	91.57	253.77

Case 130-GLIOMA	chr12:49423224	C>A	exonic	KMT2D	NM_003482 : c.G14035T	p.A4679S	42,67/43,63	50.7	49.3	326.47
Case 130-GLIOMA	chr17:36881009	C>T	exonic	MLLT6	NM_005937 : c.C3020T	p.A1007V	25,51/8,24	70.37	29.63	182.95
Case 130-GLIOMA	chr20:46265162	T>G	exonic	NCOA3	NM_181659 : c.T2032G	p.L678V	52,46/103,96	33	67	946.39
Case 130-GLIOMA	chr17:29677208	T>TA	exonic	NF1	NM_000267 : c.7266_7267insA	p.T2423Nfs	88,77/145,125	37.93	62.07	789.67
Case 130-GLIOMA	chr15:88472622	G>A	exonic	NTRK3	NM_001012338 : c.C1933T	p.R645C	37,82/56,89	45.08	54.92	642.77
Case 130-GLIOMA	chr15:88476307	C>T	exonic	NTRK3	NM_001012338 : c.G1825A	p.D609N	91,72/87,90	47.94	52.06	621.8
Case 130-GLIOMA	chr1:144881463	C>T	exonic	PDE4DIP	NM_001198834 : c.G3733A	p.A1245T	48,16/234,144	14.48	85.52	784.55
Case 130-GLIOMA	chr5:149516594	G>A	exonic	PDGFRB	NM_002609 : c.C17T	p.A6V	30,26/33,39	43.75	56.25	138.31
Case 130-GLIOMA	chr12:7067226	G>A	exonic	PTPN6	NM_080548 : c.G1357A	p.V453M	45,84/38,62	56.33	43.67	305.78
Case 130-GLIOMA	chr8:145737579	G>A	exonic	RECQL4	NM_004260 : c.C3184T	p.R1062W	48,39/47,35	51.48	48.52	246.5
Case 130-GLIOMA	chr3:128369613	G>C	exonic	RPN1	NM_002950 : c.C31G	p.L11V	26,40/14,28	61.11	38.89	174.54
Case 130-GLIOMA	chr17:78765287	G>A	exonic	RPTOR	NM_020761 : c.G868A	p.V290I	106,120/44,37	73.62	26.38	628.62
Case 130-GLIOMA	chr16:2138543	C>T	exonic	TSC2	NM_000548 : c.C5356T	p.P1786S	20,19/42,51	29.55	70.45	198.42

**ID**	**Chr:Pos**	**Base Change**	**Region**	**Gene**	**CDS**	**AA_change**	**Alt+,Alt-/Ref+,Ref-**	**%VAF**	**%RAF**	**Avg Cov**
CASE 132-GLIOMA	chr22:23654020	G>A	exonic	BCR	NM_004327 : c.G3319A	p.V1107I	26,71/160,249	19.17	80.83	674.58
CASE 132-GLIOMA	chr17:33281597	C>T	exonic	CCT6B	NM_006584 : c.G358A	p.A120T	85,217/37,81	71.9	28.1	686.68
CASE 132-GLIOMA	chr7:100421430	C>T	exonic	EPHB4	NM_004444 : c.G247A	p.A83T	56,69/60,68	49.41	50.59	263.24
CASE 132-GLIOMA	chr6:108984811	A>T	exonic	FOXO3	NM_001455 : c.A775T	p.K259X	42,46/234,265	14.99	85.01	1021
CASE 132-GLIOMA	chr5:35867528	G>C	exonic	IL7R	NM_002185 : c.G342C	p.K114N	154,114/204,123	45.04	54.96	673.08
CASE 132-GLIOMA	chr16:85945187	G>A	exonic	IRF8	NM_002163 : c.G370A	p.V124M	119,63/130,78	46.67	53.33	422.77
CASE 132-GLIOMA	chr8:41794939	G>A	exonic	KAT6A	NM_006766 : c.C3187T	p.P1063S	110,173/156,192	44.85	55.15	776.09
CASE 132-GLIOMA	chr10:129904142	G>C	exonic	MKI67	NM_002417 : c.C5962G	p.P1988A	141,163/387,458	26.46	73.54	1939.67
CASE 132-GLIOMA	chr5:79950727	GCAGCGCCCC>G	exonic	MSH3	NM_002439 : c.182_190delCAGCGCCCC	p.P67_P69del	4,76/48,160	27.78	72.22	358.86
CASE 132-GLIOMA	chr17:29560088	CA>C	exonic	NF1	NM_000267 : c.3566delA	p.G1190Afs	266,198/130,114	65.54	34.46	937.71
CASE 132-GLIOMA	chr5:176638736	C>G	exonic	NSD1	NM_172349 : c.C2529G	p.S843R	213,240/227,244	49.03	50.97	1147.75
CASE 132-GLIOMA	chr2:242045997	C>T	exonic	PASK	NM_015148 : c.G3956A	p.R1319H	59,25/158,86	25.61	74.39	387.46
CASE 132-GLIOMA	chr2:242065799	G>T	exonic	PASK	NM_015148 : c.C2531A	p.P844Q	113,97/51,44	68.85	31.15	353.56
CASE 132-GLIOMA	chr2:242065986	C>G	exonic	PASK	NM_015148 : c.G2344C	p.G782R	49,39/147,132	23.98	76.02	353.56
CASE 132-GLIOMA	chr13:49030416	C>A	exonic	RB1	NM_000321 : c.C1891A	p.Q631K	147,94/338,323	26.72	73.28	1069.43
CASE 132-GLIOMA	chrX:128605236	C>G	exonic	SMARCA1	NM_003069 : c.G2510C	p.G837A	210,167/237,223	45.04	54.96	949.4
CASE 132-GLIOMA	chr22:38374092	A>T	exonic	SOX10	NM_006941 : c.T479A	p.L160H	5,8/41,96	8.67	91.33	179.27

**ID**	**Chr:Pos**	**Base Change**	**Region**	**Gene**	**CDS**	**AA_change**	**Alt+,Alt-/Ref+,Ref-**	**%VAF**	**%RAF**	**Avg Cov**
CASE 60-GLIOMA	chrX:1522163	CA>C	exonic	ASMTL	NM_004192 : c.1864delT	p.X622Efs	208,76/14,1	94.98	5.02	787.06

CASE 60-GLIOMA	chrX:1531700	A>T	exonic	ASMTL	NM_004192 : c.T1570A	p.C524S	104,123/136,165	42.99	57.01	916.44
CASE 60-GLIOMA	chr12:113531455	C>T	exonic	DTX1	NM_004416 : c.C1115T	p.P372L	52,37/60,51	44.5	55.5	194
CASE 60-GLIOMA	chr3:10089607	A>G	exonic	FANCD2	NM_001018115 : c.A1285G	p.K429E	60,36/371,211	14.16	85.84	1297.93
CASE 60-GLIOMA	chr11:22646561	G>A	exonic	FANCF	NM_022725 : c.C796T	p.R266C	144,158/192,169	45.55	54.45	1005.19
CASE 60-GLIOMA	chr9:35076975	C>T	exonic	FANCG	NM_004629 : c.G770A	p.R257H	115,99/129,104	47.87	52.13	612.39
CASE 60-GLIOMA	chr2:216235046	C>T	exonic	FN1	NM_212482 : c.G6824A	p.R2275Q	126,110/56,37	71.73	28.27	479.43
CASE 60-GLIOMA	chr6:108882647	TCGGCGG>T	exonic	FOXO3	NM_001455 : c.237_242delCGGCGG	p.G83_G84del	39,35/73,89	31.36	68.64	221.03
CASE 60-GLIOMA	chr6:108882706	C>T	exonic	FOXO3	NM_001455 : c.C295T	p.R99W	16,56/32,83	38.5	61.5	221.03
CASE 60-GLIOMA	chr20:57429696	C>G	exonic	GNAS	NM_080425 : c.C1376G	p.P459R	21,36/9,31	58.76	41.24	233.58
CASE 60-GLIOMA	chr12:49445189	TGGCTCCTCAGGCCGGGGGGACAGGTGC>T	exonic	KMT2D	NM_003482 : c.2250_2276delGCACCTGTCCCCCGGCCTGAGGAGCC	p.R755_P763del	50,64/176,220	30.32	69.68	912.65
CASE 60-GLIOMA	chr3:65415516	C>T	exonic	MAGI1 (BAP1)	NM_001033057 : c.G1846A	p.V616M	49,50/170,194	21.38	78.62	891.56
CASE 60-GLIOMA	chr3:38181894	G>A	exonic	MYD88	NM_001172567 : c.G518A	p.R173H	126,66/64,38	65.31	34.69	603.23
CASE 60-GLIOMA	chr17:29559089	A>G	splicing	NF1	NM_000267 : c.3198-2A>G	p.?	86,27/35,15	69.33	30.67	465.85
CASE 60-GLIOMA	chr9:139417431	C>G	exonic	NOTCH1	NM_017617 : c.G613C	p.V205L	45,33/46,27	51.66	48.34	187.06
CASE 60-GLIOMA	chr2:242065799	G>T	exonic	PASK	NM_015148 : c.C2531A	p.P844Q	84,64/38,26	69.81	30.19	310.12
CASE 60-GLIOMA	chr2:242046785	A>C	exonic	PASK	NM_015148 : c.T3797G	p.F1266C	158,89/1,1	100	0	348.68
CASE 60-GLIOMA	chr1:144854574	T>C	exonic	PDE4DIP	NM_001198834 : c.A6896G	p.H2299R	65,95/463,491	14.36	85.64	1439.05
CASE 60-GLIOMA	chr8:145736909	C>A	exonic	RECQL4	NM_004260 : c.G3532T	p.G1178W	59,73/16,27	75.43	24.57	205.73
CASE 60-GLIOMA	chr7:92733601	G>A	exonic	SAMD9	NM_001193307 : c.C1810T	p.Q604X	196,196/186,180	51.72	48.28	1811.59
CASE 60-GLIOMA	chr12:54577514	G>C	exonic	SMUG1	NM_001243788 : c.C211G	p.R71G	109,129/113,154	47.13	52.87	576.61
CASE 60-GLIOMA	chr1:16260917	G>A	exonic	SPEN	NM_015001 : c.G8182A	p.A2728T	103,101/31,35	75.56	24.44	811.5
CASE 60-GLIOMA	chrX:48887947	C>T	exonic	TFE3	NM_006521 : c.G1450A	p.A484T	78,57/57,76	50.37	49.63	290.26

**ID**	**Chr:Pos**	**Base Change**	**Region**	**Gene**	**CDS**	**AA_change**	**Alt+,Alt-/Ref+,Ref-**	**%VAF**	**%RAF**	**Avg Cov**
CASE 134-GLIOMA	chr6:157099260	A>G	exonic	ARID1B	NM_020732 : c.A197G	p.N66S	9,24/15,22	47.14	52.86	92.12
CASE 134-GLIOMA	chrX:76856021	T>C	exonic	ATRX	NM_000489 : c.A5579G	p.N1860S	176,240/1,1	100	0	494.65
CASE 134-GLIOMA	chrX:129147373	G>A	exonic	BCORL1	NM_021946 : c.G625A	p.G209S	151,137/2,1	99.31	0.69	355.95
CASE 134-GLIOMA	chr9:136913474	C>T	exonic	BRD3	NM_007371 : c.G817A	p.A273T	138,137/65,63	68.24	31.76	417.76
CASE 134-GLIOMA	chr12:113515442	A>T	exonic	DTX1	NM_004416 : c.A473T	p.Q158L	59,116/93,147	42.17	57.83	655.05
CASE 134-GLIOMA	chr7:151874751	C>T	exonic	KMT2C	NM_170606 : c.G7787A	p.R2596Q	326,344/364,345	48.59	51.41	1876.38
CASE 134-GLIOMA	chr7:151882672	C>A	exonic	KMT2C	NM_170606 : c.G5053T	p.A1685S	87,63/367,305	18.25	81.75	779.12
CASE 134-GLIOMA	chr12:49445392	G>T	exonic	KMT2D	NM_003482 : c.C2074A	p.P692T	387,290/322,292	52.44	47.56	1444.84
CASE 134-GLIOMA	chr17:29667632	CT>C	exonic	NF1	NM_000267 : c.6969delT	p.L2323X	111,186/61,104	64.29	35.71	576.6
CASE 134-GLIOMA	chr6:32166766	G>A	exonic	NOTCH4	NM_004557 : c.C4472T	p.P1491L	60,49/151,144	26.98	73.02	357.22
CASE 134-GLIOMA	chr1:144855783	G>A	exonic	PDE4DIP	NM_001198834 : c.C6770T	p.A2257V	51,82/215,250	22.24	77.76	659.95

CASE 134-GLIOMA	chr7:128851596	C>G	exonic	SMO	NM_005631 : c.C1921G	p.P641A	24,55/20,69	47.02	52.98	271
CASE 134-GLIOMA	chr10:70332891	C>G	exonic	TET1	NM_030625 : c.C796G	p.P266A	291,248/140,114	67.97	32.03	806.14
CASE 134-GLIOMA	chr10:70406189	G>C	exonic	TET1	NM_030625 : c.G3703C	p.V1235L	294,287/110,101	73.36	26.64	1105.02
CASE 134-GLIOMA	chr8:15605938	C>A	exonic	TUSC3	NM_006765 : c.C992A	p.S331X	49,65/151,173	26.03	73.97	463.88

**ID**	**Chr:Pos**	**Base Change**	**Region**	**Gene**	**CDS**	**AA_change**	**Alt+,Alt-/Ref+,Ref-**	**%VAF**	**%RAF**	**Avg Cov**
CASE 194-GLIOMA	chrX:76872079	ACCTGTTAAGTGATCTAAGTAG>A	exonic	ATRX	NM_000489 : c.5547_5566+1del	p.Y1849*	69,46/320,276	16.17	83.83	649.07
CASE 194-GLIOMA	chr7:101713691	G>A	exonic	CUX1	NM_001913 : c.G295A	p.V99I	166,171/163,175	49.93	50.07	655.16
CASE 194-GLIOMA	chr3:10105516	A>C	exonic	FANCD2	NM_001018115 : c.A1868C	p.Q623P	170,197/450,288	33.21	66.79	1249.54
CASE 194-GLIOMA	chr11:118344240	C>T	exonic	KMT2A	NM_001197104 : c.C2366T	p.T789I	247,287/264,302	48.55	51.45	1452.62
CASE 194-GLIOMA	chr5:79974821	C>T	exonic	MSH3	NM_002439 : c.C1249T	p.R417W	139,192/235,273	39.45	60.55	823.9
CASE 194-GLIOMA	chr17:29588781	GC>G	exonic	NF1	NM_000267 : c.4568delC	p.A1523Dfs*30	272,215/427,408	36.84	63.16	1331.74
CASE 194-GLIOMA	chr17:29664505	CG>C	exonic	NF1	NM_000267 : c.6485delG	p.D2163Tfs*16	175,226/275,301	41.04	58.96	1087.43
CASE 194-GLIOMA	chr9:139411768	C>T	exonic	NOTCH1	NM_017617 : c.G1511A	p.R504H	117,96/121,120	46.92	53.08	387.93
CASE 194-GLIOMA	chr5:131951706	G>T	exonic	RAD50	NM_005732 : c.G3048T	p.R1016S	137,29/123,40	50.46	49.54	482.37
CASE 194-GLIOMA	chr16:2121617	T>C	exonic	TSC2	NM_000548 : c.T1946C	p.M649T	112,181/119,222	46.21	53.79	806.44
CASE 194-GLIOMA	chr9			CDKN2A/B		Homozygous loss				

**ID**	**Chr:Pos**	**Base Change**	**Region**	**Gene**	**CDS**	**AA_change**	**Alt+,Alt-/Ref+,Ref-**	**%VAF**	**%RAF**	**Avg Cov**
CASE 351-GLIOMA	chr12:52385751	A>G	exonic	ACVR1B	NM_020328.3 : c.A1489G	p.I497V	350,355/602,699	35.14	64.86	2717.92
CASE 351-GLIOMA	chr20:31024254	C>T	exonic	ASXL1	NM_015338.5 : c.C3739T	p.R1247C	254,298/255,310	49.42	50.58	1984.45
CASE 351-GLIOMA	chrX:76875920	G>A	exonic	ATRX	NM_000489.4 : c.C5215T	p.R1739*	109,170/61,72	67.72	32.28	417.67
CASE 351-GLIOMA	chr6:18249892	C>T	exonic	DEK	NM_003472.3 : c.G752A	p.S251N	167,10/339,38	31.95	68.05	1362.75
CASE 351-GLIOMA	chr6:15496952	C>T	exonic	JARID2	NM_004973.3 : c.C1496T	p.P499L	295,250/288,245	50.56	49.44	1358.12
CASE 351-GLIOMA	chr17:29541603	TGTAA>T	splicing	NF1	NM_000267 : c.1527+1_1527+4delGTAA	p.?	112,227/59,75	71.67	28.33	551.35
CASE 351-GLIOMA	chr17:5462523	C>T	exonic	NLRP1	NM_033004.3 : c.G1493A	p.R498K	213,231/363,386	37.22	62.78	1799.03
CASE 351-GLIOMA	chr7:6018314	TGAGAGT>TGAGC	exonic	PMS2	NM_000535.6 : c.2182_2187delinsGCTC	p.L728Afs*6	42,110/512,474	13.36	86.64	1034.43
CASE 351-GLIOMA	chr7:6035200	A>C	exonic	PMS2	NM_000535.6 : c.T868G	p.F290V	541,308/355,184	61.17	38.83	1723.8
CASE 351-GLIOMA	chr7:103216152	G>C	exonic	RELN	NM_005045.3 : c.C4146G	p.S1382R	153,310/194,326	47.1	52.9	1561.11
CASE 351-GLIOMA	chr7:103275891	G>A	exonic	RELN	NM_005045.3 : c.C2446T	p.L816F	283,107/310,148	45.99	54.01	1328.6
CASE 351-GLIOMA	chrX:70601712	C>T	exonic	TAF1	NM_004606.4 : c.C1540T	p.R514W	281,472/1,1	99.74	0.26	1270.94
CASE 351-GLIOMA	chr10:98133376	C>T	exonic	TLL2	NM_012465.3 : c.G2639A	p.G880D	174,57/236,95	41.1	58.9	838.43
CASE 351-GLIOMA	chr16:2138570	C>T	exonic	TSC2	NM_000548.4 : c.C5383T	p.R1795C	114,185/121,200	48.23	51.77	756.3
CASE 351-GLIOMA	chr9			CDKN2A/B		Homozygous loss				

Table S3. Telomere alterations in rare NF1-associated tumors (n=46).

Tumor Type	N	Telomere Abnormalities (%)				ATRX alterations
		ALT	Short	Long	Normal	Loss by IHC
GIST	12	0	5 (42%)	0	7 (58%)	0 (of 6)
Pheochromocytoma	7	0	1 (100%)	0	0	1 (of 4)
Glomus tumor	5	0	0	0	4 (100%)	0 (of 4)
Neuroendocrine tumors	6	0	4 (66%)	0	2 (33%)	No *ATRX/DAXX* mutations[28]
Lipoma	5	0	0	0	4 (100%)	-
Schwannoma	2	0	0	0	2 (100%)	0 (of 2)
Juvenile xanthogranuloma	3	0	1 (33%)	0	2 (66%)	0 (of 3)
Gangliocytic paraganglioma	2	0	0	0	1 (100%)	-
Dermatofibroma	1	0	0	0	1 (100%)	-
Ovarian serous carcinoma	1	0				0 (of 1)
Syringocystadenoma papilliferum	1	0	0	0	1 (100%)	0 (of 1)
Malignant phyllodes tumor	1	1	0	0	0	0 (of 1)

[28]Noë et al, Modern Pathology, 2018.

Table S4. Association of telomere length with overall survival in patients with glioma.

			Univariate*			Multivariate		
	Death/Censor	Person-time (months)	HR	(95% CI)	P-value	HR	(95% CI)	P-value
**Age**	15/17	2068	1.05	(1.01-1.09)	0.03	1.02	(0.95-1.08)	0.6
**Grade**								
Low	3/12	1813	1	(Ref)	-	1	(Ref)	-
High	12/15	255	7.36	(2.18-24.77)	0.001	3.09	(0.70-13.60)	0.1
**Telomere Length**								
Normal	3/10	1392	1	(Ref)	-	1	(Ref)	-
Long	4/3	428	3.99	(0.70-22.77)	0.1	3.2	(0.47-21.92)	0.2
ALT	8/4	248	13.05	(2.35-72.43)	0.003	4.16	(0.40-42.88)	0.2

*Cases without telomere length are not included.

Table S5. Association of telomere lengths with overall survival in all patients with MPNST

			Univariate			Multivariate		
	Death/Censor	Person-time (months)	HR	(95% CI)	P-value	HR	(95% CI)	P-value
**Age**	24/25	1779	1.01	(0.99-1.03)	0.4	1.01	(0.99-1.03)	0.4
**Grade**								
Low	1/3	168	1.00	(Ref)	-	1.00	(Ref)	-
High	23/22	1612	2.86	(0.38-21.40)	0.3	3.48	(0.42-28.97)	0.2
**Telomere lengths**								
Normal	6/10	417	1.00	(Ref)	-	1.00	(Ref)	-
Short	1/13	801	0.13	(0.02-1.07)	0.06	0.09	(0.01-0.79)	0.03
ALT	17/2	561	1.95	(0.76-5.00)	0.2	1.30	(0.43-3.91)	0.6

Table S6. Association of ALT with overall survival in all patients with MPNST.

			Univariate*			Multivariate		
	**Death/Censor**	**Person-time (months)**	**HR**	**(95% CI)**	**P-value**	**HR**	**(95% CI)**	**P-value**
**Age**	76/51	3818	1.01	(0.99-1.02)	0.3	1.01	(0.99-1.02)	0.3
**Grade**								
Low	3/10	688	1.00	(Ref)	-	1.00	(Ref)	-
High	73/41	3130	5.47	(1.71-17.46)	0.004	5.32	(1.66-17.07)	0.005
**ALT status**								
Negative	59/49	3256	1.00	(Ref)	-	1.00	(Ref)	-
Positive	17/2	561	1.51	(0.87-2.60)	0.1	1.23	(0.70-2.15)	0.5

*Cases without ALT status are not included.
